# SETD4-expressing cells contribute to pancreatic development and response to cerulein induced pancreatitis injury

**DOI:** 10.1038/s41598-021-92075-5

**Published:** 2021-06-15

**Authors:** Jin-Ze Tian, Sheng Xing, Jing-Yi Feng, Shu-Hua Yang, Yan-Fu Ding, Xue-Ting Huang, Jin-Shu Yang, Wei-Jun Yang

**Affiliations:** 1 MOE Laboratory of Biosystem Homeostasis and Protection, College of Life, Sciences, Zhejiang University, Hangzhou, 310058 China; 2 Laboratory for Marine Biology and Biotechnology, Qingdao National Laboratory for Marine Science and Technology, Qingdao, 266000 China

**Keywords:** Cell biology, Developmental biology

## Abstract

In the adult pancreas, the presence of progenitor or stem cells and their potential involvement in homeostasis and regeneration remains unclear. Here, we identify that SET domain-containing protein 4 (SETD4), a histone lysine methyltransferase, is expressed in a small cell population in the adult mouse pancreas. Genetic lineage tracing shows that during pancreatic development, descendants of SETD4^+^ cells make up over 70% of pancreatic cells and then contribute to each pancreatic lineage during pancreatic homeostasis. SETD4^+^ cells generate newborn acinar cells in response to cerulein-induced pancreatitis in acinar compartments. Ablation of SETD4^+^ cells compromises regeneration of acinar cells, in contrast to controls. Our findings provide a new cellular narrative for pancreatic development, homeostasis and response to injury via a small SETD4^+^ cell population. Potential applications may act to preserve pancreatic function in case of pancreatic disease and/or damage.

## Introduction

The pancreas is composed of two morphologically and functionally distinct components: the endocrine pancreas (islets of Langerhans) and the exocrine pancreas (acinar cells and ductal cells). Its endocrine function largely concerns insulin and the regulation of blood sugar whereas its exocrine function relates to the production of digestive enzymes. Exocrine acinar cells produce an array of digestive enzymes, secreted into pancreatic ducts and then flowing into the small intestine to break down fats, proteins, and carbohydrates for absorption^1^. The exocrine pancreas possesses an intrinsic capacity for regeneration and thus can make a rapid and full recovery from exocrine diseases such as acute pancreatitis^2–5^. By contrast, the endocrine islets have limited regenerative capacity in adults^6^. The current explanation for the regenerative capacities of the exocrine pancreas is that acinar cells in the adult pancreas show a high degree of plasticity and can undergo trans-differentiation to a progenitor-like cell type with ductal characteristics^7–9^. This process is considered an important feature facilitating pancreatic regeneration after injury. Genetic lineage tracing has shown that the major mechanism for β cell replenishment in homeostasis or after injury was replication of pre-existing β cells^10^. Other reports have demonstrated that the origin of β cells during responses to injury include endocrine α cells, β cells, neogenesis from the duct epithelium or acini-to-endocrine or acini-to-duct-to-endocrine trans-differentiation^11–15^.

Previous studies have also shown that all pancreatic cell types are derived from a pool of early pancreatic progenitor cells that express the transcription factors Pdx1, Ptf1a, Sox9^16–19^. In the mouse, pdx1 expression begins at E8.0^20,21^, prior to the onset of pancreatic bud formation and islet hormone gene expression, and is initially detected throughout the pancreatic epithelium. By late gestation, pdx1 expression is selectively maintained at high levels in β cells, with low levels of expression in acinar cells^20,22^. Several mouse and human based studies have shown that the loss of pdx1 function results in an early block in pancreatic outgrowth and differentiation^23–26^. Sox9 is a members of the SRY/HMG box (Sox) family of transcription factors^27–29^. Akiyama et al. used transgenic mice carrying lacZ inserted into the Sox9 gene and identified Sox9 as expressed in the adult pancreatic ductal epithelium. Lineage tracing in the same study revealed that pancreatic cells from all lineages had been derived from Sox9-expressing precursors^16^. In addition, the β-cell-restricted transcription factor Nkx6.1 is also noted as essential for maintaining the functional state of β-cells during adulthood^30^. Both in vitro and in vivo experiments have suggested an important role for Nkx6.1 in β-cell proliferation^30–32^. However, the mechanism of the establishment of stem/progenitor cells for pancreas homeostatic and injury-induced regeneration currently remains unclear.

One explanation for regenerative capacities of the pancreas has been suggested to be the presence of quiescent or reserve progenitors^33^. Examples of quiescent cell populations include hematopoietic stem cells, hair follicle stem cells, intestinal stem cells, muscle stem cells, neural stem cells and cancer stem cells^34–38^. Cellular quiescence, as a reversible nondividing state and the counterpoint to proliferation, is a conserved mechanism that occurs in somatic stem cells^39,40^. Previous studies in various types of tissues have identified that these quiescent cells can contribute to the long-term maintenance of a stem cell pool by preserving proliferation capacity and acting as a cell reservoir. They then facilitate tissue homeostasis and regeneration in response to tissue injury upon activation^41,42^.

Our previous studies have shown SET domain-containing protein 4 (SETD4) as abundantly expressed in Artemia dormant embryos, and that SETD4-defined quiescent cancer stem cells were resistant to chemoradiotherapy and able to produce a cancer cell population upon activation in breast cancer cells^43,44^. We established an evolutionarily conserved mechanism of cell quiescence, in which SETD4 epigenetically controls cell quiescence by facilitating heterochromatin formation via the trimethylation of lysine 20 of histone 4 (H4K20me3) catalysis^44^. Upon our confirmation that SETD4 also marks quiescent cells in the adult mouse pancreas, we then began to examine to what extent SETD4^+^ cells contribute to pancreatic development and regeneration. In this study, we show that SETD4^+^ cells contribute to pancreatic development and homeostatic regeneration by contributing to each pancreatic lineage. SETD4^+^ cells generate newborn acinar cells in response to cerulein-induced pancreatitis in acinar compartments. In addition, the ablation of SETD4^+^ cells compromised the regeneration of acinar cells, in contrast to controls. Our results reveal that SETD4^+^ cells make a significant contribution to pancreatic development and response to pancreatic injury in adults.

## Results

### Identification and characterization of SETD4-expressing cells in the adult mouse pancreas

**Figure 1:**
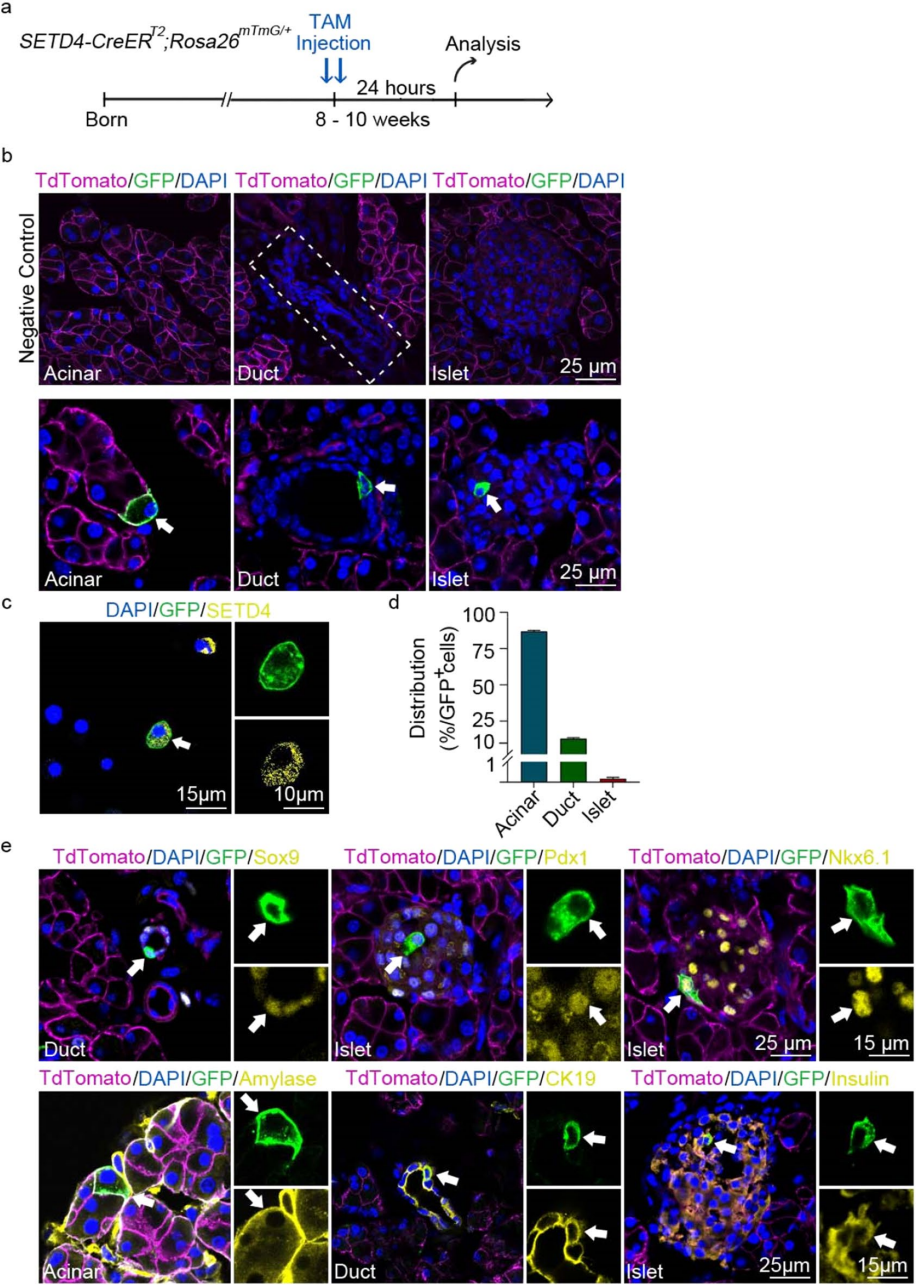
Identification of SETD4^+^ cells in the adult pancreas of *SETD4-CreER*^*T2*^;*Rosa26*^*mTmG/*+^ mice. (**a**) Schedule of 24-h TAM-induction for SETD4^+^ cells identification in adult *SETD4-CreER*^*T2*^*;Rosa26*^*mTmG/*+^mice. (**b**) Representative fluorescent images of negative control of non-TAM induction and recombinant GFP^+^ cells in acinar, ducts and islets 24 h after TAM-induction. Scale bar for each image, 25 μm. (**c**) Representative immunofluorescence for identification of GFP^+^ cells in pancreas using an anti-SETD4 antibody by immunocytofluorescence. Scale bar for merged images, 15 μm. Scale bar for all spilt images, 10 μm. (**d**) Quantification of recombinant GFP^+^ cells (SETD4^+^ cells) in acinar, duct and islet compartments. Recombination was assessed 24 h after TAM treatment with 80–100 high power fields randomly selected. Recombinant cells were counted manually and divided into acinar, duct and islet cells based on their respective morphology. (**e**) Representative immunofluorescence for recombinant (SETD4^+^) cells with amylase in the acinar, Sox9 and CK19 in the ducts, Pdx1, Nkx6.1 and insulin in the islets in adult *SETD4-CreER*^*T2*^*;Rosa26*^*mTmG/*+^mice. Scale bar for merged images, 25 μm. Scale bar for all spilt images, 15 μm.

In this study, we inserted *CreER*^*T2*^ into the *SETD4* locus, and crossed *SETD4-CreER*^*T2*^ with *Rosa26*^*mTmG/*+^ to generate *SETD4*-*CreER*^*T2*^;*Rosa26*^*mTmG/*+^ transgenic mice (Fig. S1a). To identify SETD4 expressing (SETD4^+^) cells in adult mouse pancreas, we performed short period Tamoxifen (TAM) induction for 24 h and then detected the recombinant of green fluorescent protein (GFP) in the pancreas of 8—10 week old mice (Fig. 1a). A small population of GFP^+^ cells were observed in the acinar, duct and islet compartments that were considered to be SETD4^+^ cells, while as a control, no recombinant GFP was detected in the absence of TAM-induction and in wide type mice (Fig. 1b, S1b). In addition, pancreatic cells were dissociated from *SETD4-CreER*^*T2*^*;Rosa26*^*mTmG/*+^ mice 24 h after TAM-induction and then fixed on glass slides. In these, over 90% GFP^+^ cells were confirmed via an anti-SETD4 antibody to express SETD4 (Fig. 1c). This result confirms that beyond 24 h TAM-induction, the GFP^+^ cells in the mice are SETD4^+^ cells. However, we failed to validate the same result on pancreatic sections using the same anti-SETD4 antibody.

We isolated SETD4^+^ (GFP^+^) cells in each compartment from adult *SETD4-CreER*^*T2*^;*Rosa26*^*mTmG/*+^ mice 24 h after TAM-induction by flow cytometry analysis (Fig. S1c). Results showed that SETD4^+^ cells were distributed in the acinus, ducts and islets of Langerhan compartments at approximately 2.24%, 0.85% and 0.27%, respectively and that the majority of SETD4^+^ cells (86.97%) were located in the acinar, with 12.94% in the ducts, with only a minority (0.09%) found in the islet compartment (Fig. 1d). In addition, we found that these SETD4^+^ cells also expressed amylose (95.8 ± 2.6%) in the acinar, Sox9 (89.6 ± 5.7%) and CK19 (88.7 ± 7.0%) in the ducts, and Pdx1 (87 ± 4.4%), Nkx6.1 (84.4 ± 4.2%) and insulin (96.4 ± 4.2%) in the islets (Fig. 1e). However, glucagon, somatostatin, and ghrelin, the corresponding markers of α, δ, and ε cells, remained undetected in SETD4^+^ cells in the islet compartment (Fig. S1d). Analysis of quantitative real-time PCR showed that the expression levels of *SETD4*, *Sox9*, *Pdx1* were significant higher and *Dclk1* significantly lower in FACS-sorted GFP^+^ (SETD4^+^) cells, in contrast to GFP^-^ (SETD4^-^) cells (Fig. S1e). This indicated that SETD4^+^ cells are distinct from SETD4^-^ pancreatic cells. A small population of SETD4^+^ cells were thereby confirmed in the adult mouse pancreas.

### SETD4^+^ cells are present in embryonic pancreas and contribute to pancreatic development

**Figure 2:**
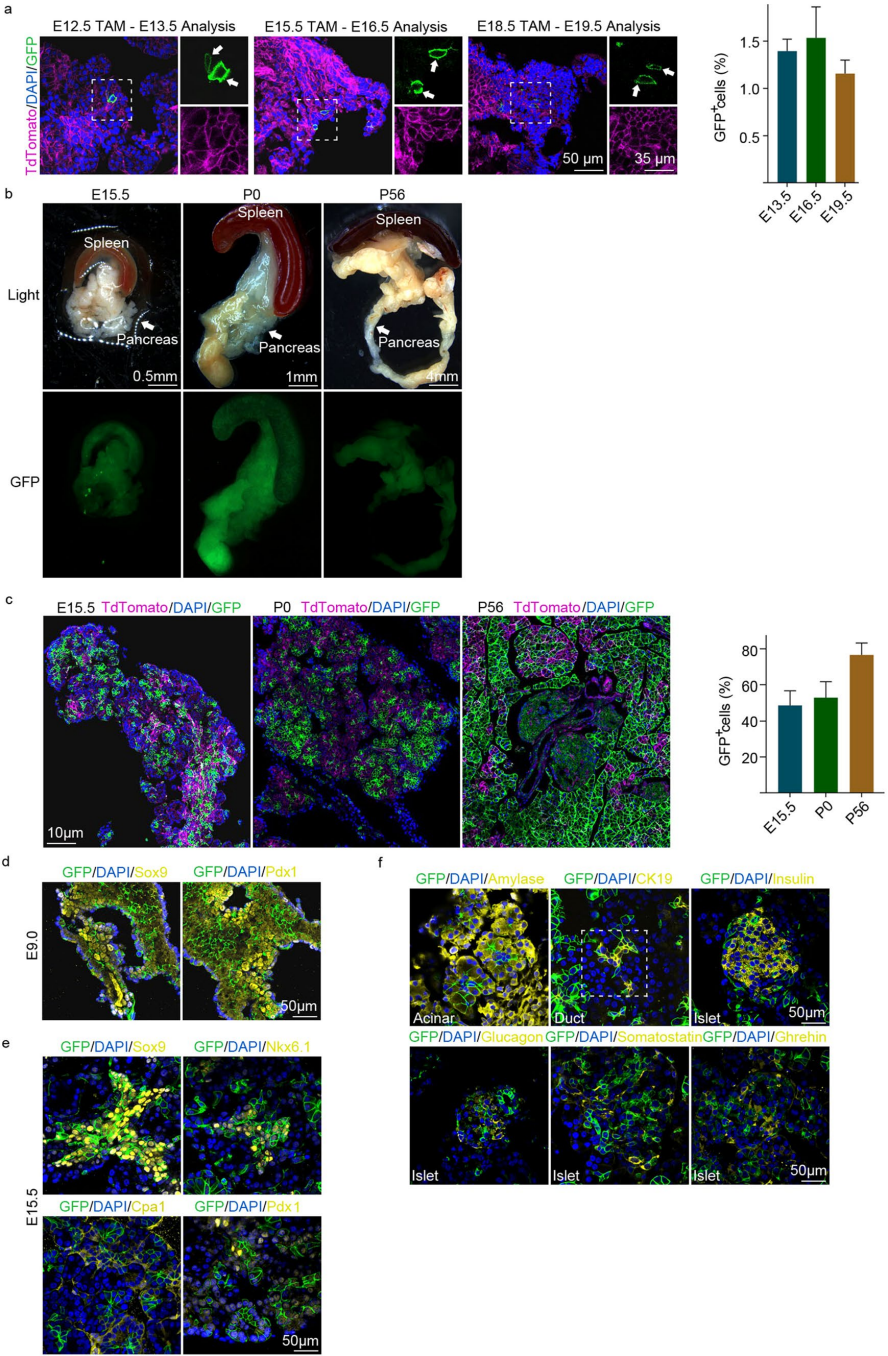
Identification of SETD4^+^ cells in embryonic *SETD4-CreER*^*T2*^;*Rosa26*^*mTmG/*+^ mice (**a**) and lineage tracing of SETD4^+^ cells in *SETD4-Cre*;*Rosa26*^*mTmG/*+^ mice (**b**–**f**). (**a**) Representative immunofluorescence and quantification for recombinant (SETD4^+^) cells 24 h after tamoxifen-induction of at embryo day 13.5 (E13.5), E16.5 and E19.5 mouse pancreases. (**b**) Representative wholemounts fluorescent images of pancreases in E15.5, P0 and P56 mice. Scale bar for each image, 0.5, 1, 4 mm, respectively. The white arrow indicates the pancreas. (**c**) Representative immunofluorescence and quantification of GFP^+^ recombinant cells in the total (DAPI^+^) cells of E15.5, P0 and P56 pancreas. Scale bar for each image, 10 μm. (**d**) Representative immunofluorescence for SETD4^+^ cells descendants (recombinant cells) with Sox9 and Pdx1 in E9.0 pancreatic bud. Scale bar for each image, 50 μm. (**e**) Representative immunofluorescence for SETD4^+^ cells descendants (recombinant cells) with Sox9, Nkx6.1, Cpa1 and Pdx1 in the E15.5 pancreas. Scale bar for each image, 50 μm. (**f**) Representative immunofluorescence for recombinant cells with amylase in the acinar, CK19 in the duct and insulin, glucagon, somatostatin and ghrelin in the islet in P56 pancreas. All data are represented as mean ± SD. n = 3 mice. Nuclei were stained with DAPI.

24 h after TAM-induction, GFP^+^ cells were detected in the embryonic pancreas of *SETD4-CreER*^*T2*^;*Rosa26*^*mTmG/*+^ mice at E13.5, E16.5 and E19.5, respectively (Fig. 2a). This result indicated SETD4^+^ cells as also present in the embryonic pancreas. To identify the lineage of SETD4^+^ cells during pancreatic development, we created *SETD4-Cre*;*Rosa26*^*mTmG/*+^ mice (Fig. S2a), in which all SETD4^+^ cells and their descendants could be observed by detection of GFP. We found that, as descendants of SETD4^+^ cells, GFP^+^ cells had distributed into the whole pancreas and then comprised over 49.7%, 53% and 77% of total cells in E15.5, P0 and P56 mice, respectively (Fig. 2b,c). While as a control, no recombinant GFP was detected in E9.0 whole embryo which from *SETD4-Cre* mice crossed with wide type mice (Fig. S2b). In addition, we found that GFP^+^ cells also expressed Sox9 and Pdx1 in the pancreatic buds at E9.0 and Sox9, Pdx1, Cpa1 and Nkx6.1 in E15.5 pancreases (Fig. 2d,e). Descendants of SETD4^+^ cells were also confirmed to contribute to each of the 3 pancreatic lineages by detection of amylase in the acinar, CK19 in the duct, and insulin, glucagon, somatostatin and ghrelin in the islet in P0 (Fig. S2c) and P56 pancreas (Fig. 2f). Taken together, these results indicate SETD4^+^ cells as present in the embryonic pancreas and confirmed their contribution to pancreatic development via the production of each lineage.

### SETD4^+^ cells generate the 3 pancreatic lineages during homeostasis in the adult

**Figure 3:**
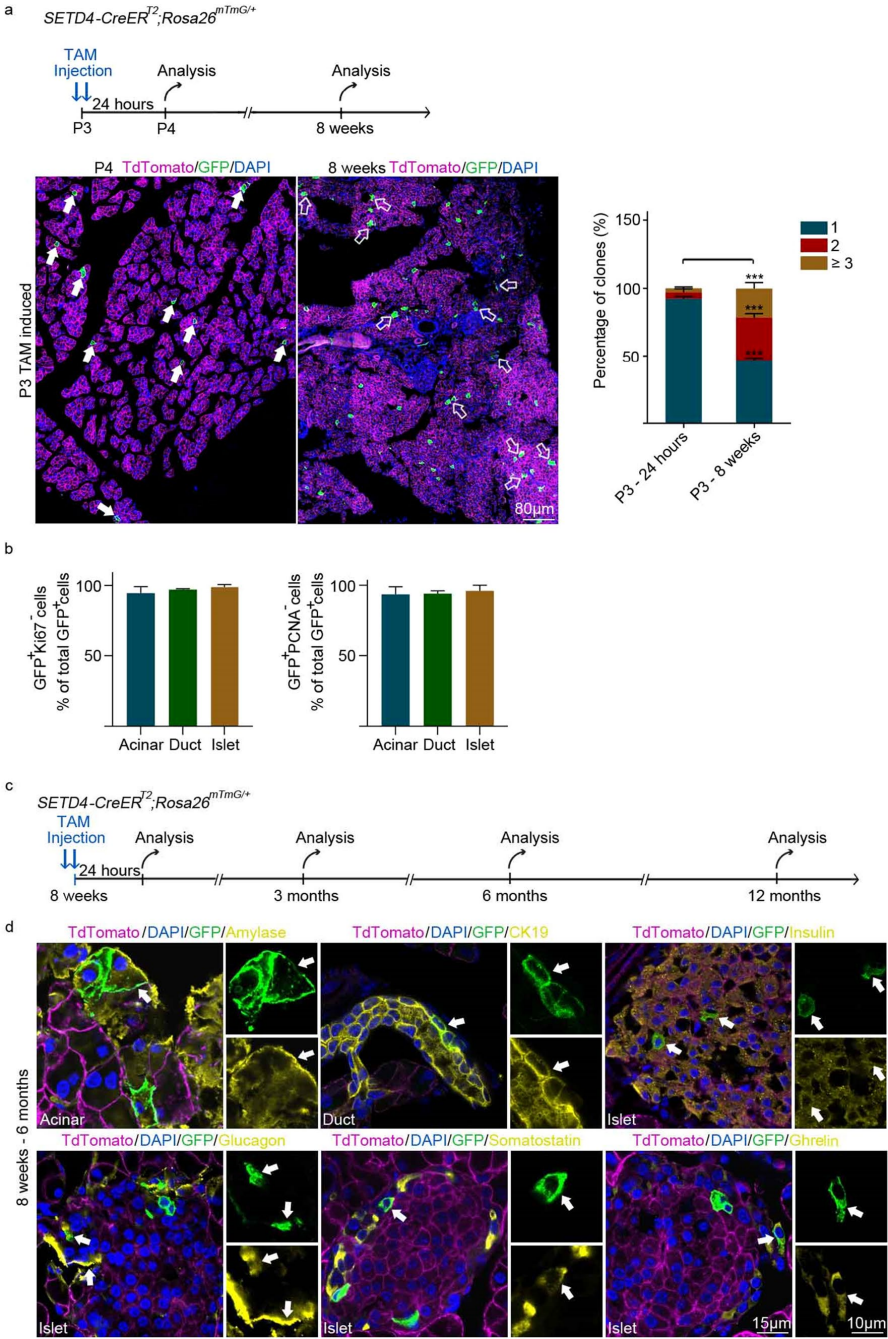

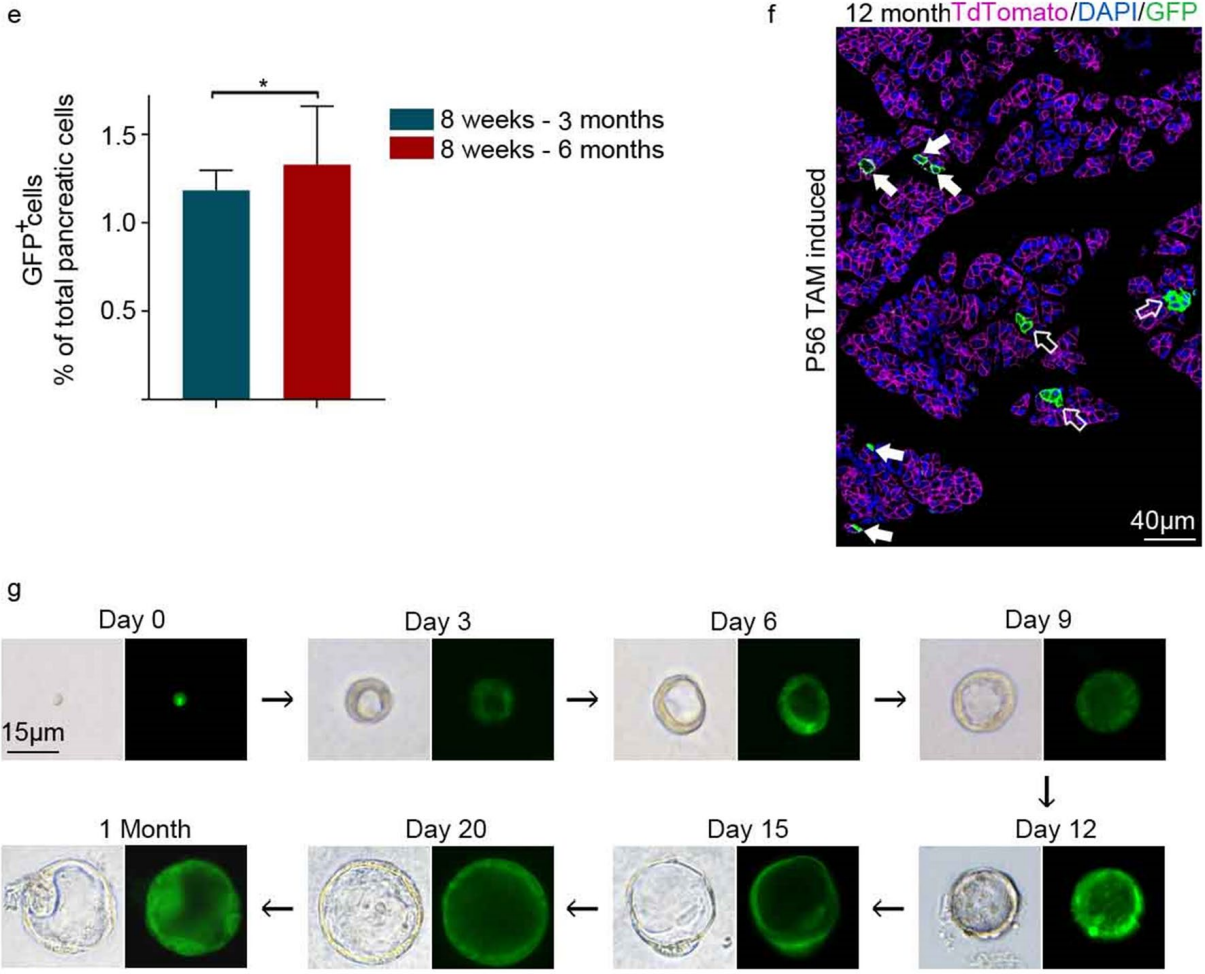
Lineage tracing of SETD4^+^ cells in P3 and 8 weeks *SETD4-CreER*^*T2*^;*Rosa26*^*mTmG/*+^ mice. (**a**) Schedule and lineage tracing of SETD4^+^ cells in P3 *SETD4-CreER*^*T2*^;*Rosa26*^*mTmG/*+^ mice. Neonatal mice were treated with 2 consecutive doses of TAM and then analyzed at 2 time points: 24 h or 8 weeks post-induction with TAM. Representative immunofluorescence and quantification of single (1), doublet (2), and clones of multi-cells (≥ 3) at the respective time points are shown. Solid arrows: single cell (1), hollow arrows: multi-cell (≥ 3) clones. Scale bar in the lineage, 80 μm. (**b**) Quantification for Ki67 and PCNA of GFP^+^ recombinant cells in the acinar, duct or islet compartments from adult pancreases. (**c**) Schedule of lineage tracing of SETD4^+^ cells in 8 weeks *SETD4-CreER*^*T2*^;*Rosa26*^*mTmG/*+^ mice. Adult mice were treated with 2 consecutive doses of TAM and then analyzed at 4 time points: 24 h, 1, 4 and 10 months post-induction with TAM. (**d**) Representative immunofluorescence for amylase, CK19, insulin, glucagon, somatostatin and ghrelin in pancreas of adult (8 weeks) mice pancreases at 4 months after TAM-induction. Scale bar for all merged images, 15 μm. Scale bar for all spilt images, 10 μm. (**e**) Quantification of recombinant GFP^+^ cells of total (DAPI^+^) pancreatic cells in adult (P56) mice 1 month and 4 months after TAM-induction. (**f**) Representative immunofluorescence of lineage tracing of SETD4^+^ cells 10 months after TAM-induction in adult *SETD4-CreER*^*T2*^;*Rosa26*^*mTmG/*+^ mice. Solid arrows: single cell (1), hollow arrows: multi-cell (≥ 3) clones. Scale bar in the lineage, 40 μm. (**g**) Isolation by fluorescence activated cell sorting (FACS) of GFP^+^ and GFP^-^ cells from *SETD4-CreER*^*T2*^;*Rosa26*^*mTmG/*+^ mice at 24 h after TAM-induction. GFP^+^ and GFP^-^ cells in duct compartment were sorted and then cultured in 3-Dimentional Matrigel. Representative formation of cell-organized organoids by SETD4^+^ cells (green) in a 3-Dimentional Matrigel-based culture over a month. Scale bar for each image, 15 μm. All data are represented as mean ± SD. *p < 0.05. ***p < 0.001. *ns* not significant. n = 4 mice. Arrows indicate recombinant GFP^+^ cells (green). Nuclei were stained with DAPI.

Next, SETD4^+^ cells in neonatal *SETD4-CreER*^*T2*^;*Rosa26*^*mTmG/*+^ mice were also identified as single-cells in the acinar, duct and islet compartments of pancreas at P3 by 24 h TAM-induction and the descendants of SETD4^+^ cells were observed with significant increases of multi-cellular clones in the 3 compartments at 8 weeks after TAM-induction (Fig. 3a). These results indicated that SETD4^+^ cells produce their descendants during pancreatic expansion in neonates. In addition, we found that 24 h after TAM-induction, SETD4^+^ cells in adult (8 weeks) *SETD4-CreER*^*T2*^;*Rosa26*^*mTmG/*+^ mice lacked the expression of the cell proliferation makers, Ki67 and PCNA in all 3 compartments, and no differences were found between these and SETD4^-^ cells. This indicated that SETD4^+^ cells were in a slow-cycling or quiescent state (Fig. 3b).

To examine whether SETD4^+^ cells in the adult pancreas produce each mature pancreatic cell for homeostasis, 1 and 4 months of TAM-induction were performed in adult (P56) *SETD4-CreER*^*T2*^;*Rosa26*^*mTmG/*+^ mice (Fig. 3c). Detection of corresponding markers showed that SETD4^+^ cells could produce all 3 lineages of acinar, duct and islet (β, α, δ, and ε) cells after 1 or 4 months (Fig. S3a, 3d). We found that, as descendants of SETD4^+^ cells, GFP^+^ cells increased over the time (Fig. 3e). Results indicated that SETD4^+^ cells had contributed to homeostatic generation in adults by producing each of the 3 lineage cells. We also found that most SETD4^+^ cells (GFP^+^ cells) had persisted as single cells for 10 months, beyond which a small population of clusters of recombinant GFP^+^ cells then appeared. This indicates that SETD4^+^ cells are long-lived and maintain their stability for 10 months under homeostatic conditions (Fig. 3f).

Organoids are self-organized three-dimensional cell cultures that contain some of the cell types and key features of the organs they represent^45,46^. They have been used to study the characteristics of adult pancreatic progenitor cells^47^. In this study, we analyzed the potential for SETD4^+^ cells to form cell-organized organoids in vitro, particularly whether they would provide a model for key features of adult pancreatic progenitors. 24 h after TAM-induction, SETD4^+^ (GFP^+^) cells were isolated from the ducts (Fig. S3b) and cultivated in a 3-Dimentional Matrigel-based culture at a seeding density of 100 cells per well. Organoids were counted on the third day after seeding. The organoid formation efficiency of SETD4^+^ cells (GFP^+^) was 42.3 ± 7.8% and they passaged at least 5 generations over 30 days (Fig. 3g, S3c). Of note, 1.1 ± 0.5% of the SETD4^-^ (GFP^-^) cells also grew into organoids. In addition, Sox9^+^, Pdx1^+^ and CK19^+^ cells were identified in the organoids, (Fig. S3d). This indicated that SETD4^+^ cells have a considerable proliferative potential and are able to self-organize into pancreatic organoids in vitro.

### SETD4^+^ cells produce newborn acinar cells in response to cerulein-induced pancreatitis

**Figure 4:**
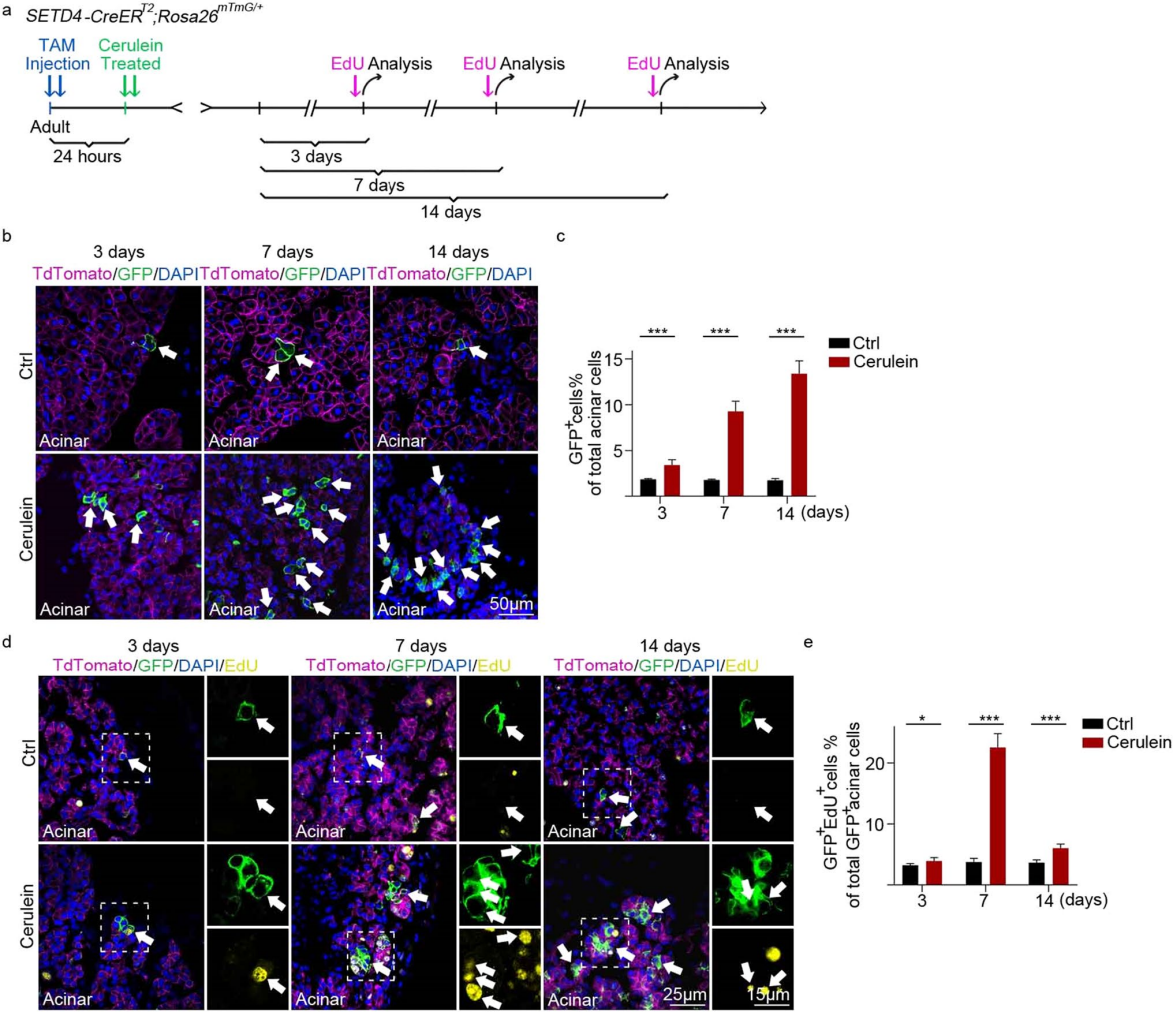
Lineage tracing of SETD4^+^ cells after cerulein-induced injury. (**a**) Experimental outline of lineage tracing of SETD4^+^ cells in adult *SETD4-CreER*^*T2*^;*Rosa26*^*mTmG/*+^ mice after Cerulein-induced injury. 8–10 week mice were subjected to an episode of experimental chronic pancreatitis to induced chronic pancreatic damage at 24 h after TAM induction. Control mice received comparable injections of 0.9% saline buffer. Mice were sacrificed at the given times: 3, 7 and 14 days after Cerulein treatment. 5-ethynyl-2’-deoxyuridine (EdU) solution was injected into mice 2 h before each collected sample at each point in time. (**b**) Representative immunofluorescence for recombinant GFP^+^ cells after 3, 7, or 14 days of Cerulein treatment and of Ctrls. Ctrl: Control. Scale bar for each image, 50 μm. (**c**) Quantification of GFP^+^ cells in acinar compartment after 3, 7, or 14 days of Cerulein treatment and the Ctrl. Ctrl: Control. All data are represented as mean ± SD. ***p < 0.001. Ctrl: Saline buffer (n = 3 mice), Cerulein (n = 4 mice). (**d**) Representative immunofluorescence for EdU staining in the acinar compartment after 3, 7, or 14 days of treatment. Ctrl: Control. Scale bar for all merged images, 25 μm. Scale bar for all spilt images, 15 μm. (**e**) Quantification of GFP^+^EdU^+^ cells in acinar compartment after 3, 7, or 14 days of treatment. Ctrl: Control. All data are represented as mean ± SD. *p < 0.05. ***p < 0.001. Ctrl: Saline buffer (n = 3 mice), Cerulein (n = 4 mice).

Mouse models of chronic pancreatitis have revealed that the exocrine pancreas possesses remarkable regenerative capacity after cerulein-induced inflammatory damage^48,49^. To investigate the function of SETD4^+^ cells in response to injury of acinar cells in the pancreas, adult *SETD4-CreER*^*T2*^;*Rosa26*^*mTmG/*+^ mice were injected with cerulein after TAM-induction (Fig. 4a). 8 to 10-week mice were randomly divided into groups of 4–6 animals and injected with TAM twice over a 24-h period. The mice were then fasted for 12 h and subjected to an episode of experimental chronic pancreatitis to induced chronic pancreatitis damage. Control mice received comparable injections of 0.9% saline buffer. Mice were sacrificed at one of three time points: 3, 7 or 14 days after treatment. 5-ethynyl-2’-deoxyuridine (EdU) solution was injected in mice 2 h before sample collection at each time point. Apoptosis of pancreatic acinar cells was identified using a terminal-deoxynucleotidyl-transferase-mediated dUTP nick-end labeling (TUNEL) assay. We observed that the apoptotic index was markedly increased at day 3 and this increase began to decrease at 7 or 14 days after cerulein induction, in comparison with the controls (Fig. S4a, S4b). By analyses of histology, the ratio of pancreas to body weight and the pancreatitis score, significant pancreatic damage to the acinar was confirmed after 3 days of cerulein treatment with repair in evidence after 7 days and completed by day 14 of treatment (Fig. S4c to S4e).

Strikingly, we found that the cerulein-induced pancreatitis led to a significant expansion of GFP^+^ acinar cells with approximately 1.84-, 5.17-, and 7.59-fold increases after 3, 7 or 14 days of treatment, respectively, in contract to controls (Fig. 4b,c). However, this expansion was not observed in the duct and islet compartments after cerulein treatment (Fig. S4f to S4i). This increase in newborn acinar cells was confirmed in the 7th day after cerulein treatment by analysis of incorporation of 5’-ethynyl-2’-deoxyuridine (GFP^+^EdU^+^) and expression of Ki67 (GFP^+^Ki67^+^) (Fig. 4d,e, S4j, S4k). Approximately 67% of Ki67^+^ and 66.1% of EdU^+^ acinar cells were also GFP^+^ (> 2,500 cells, n = 3) (Fig. S4l). We concluded that SETD4^+^ cells predominantly contribute to pancreatic regeneration by producing proliferative newborn acinar cells in response to cerulein-induced pancreatitis.

### Ablation of SETD4^+^ cells failed to repair cerulein induced pancreatitis damage

**Figure 5:**
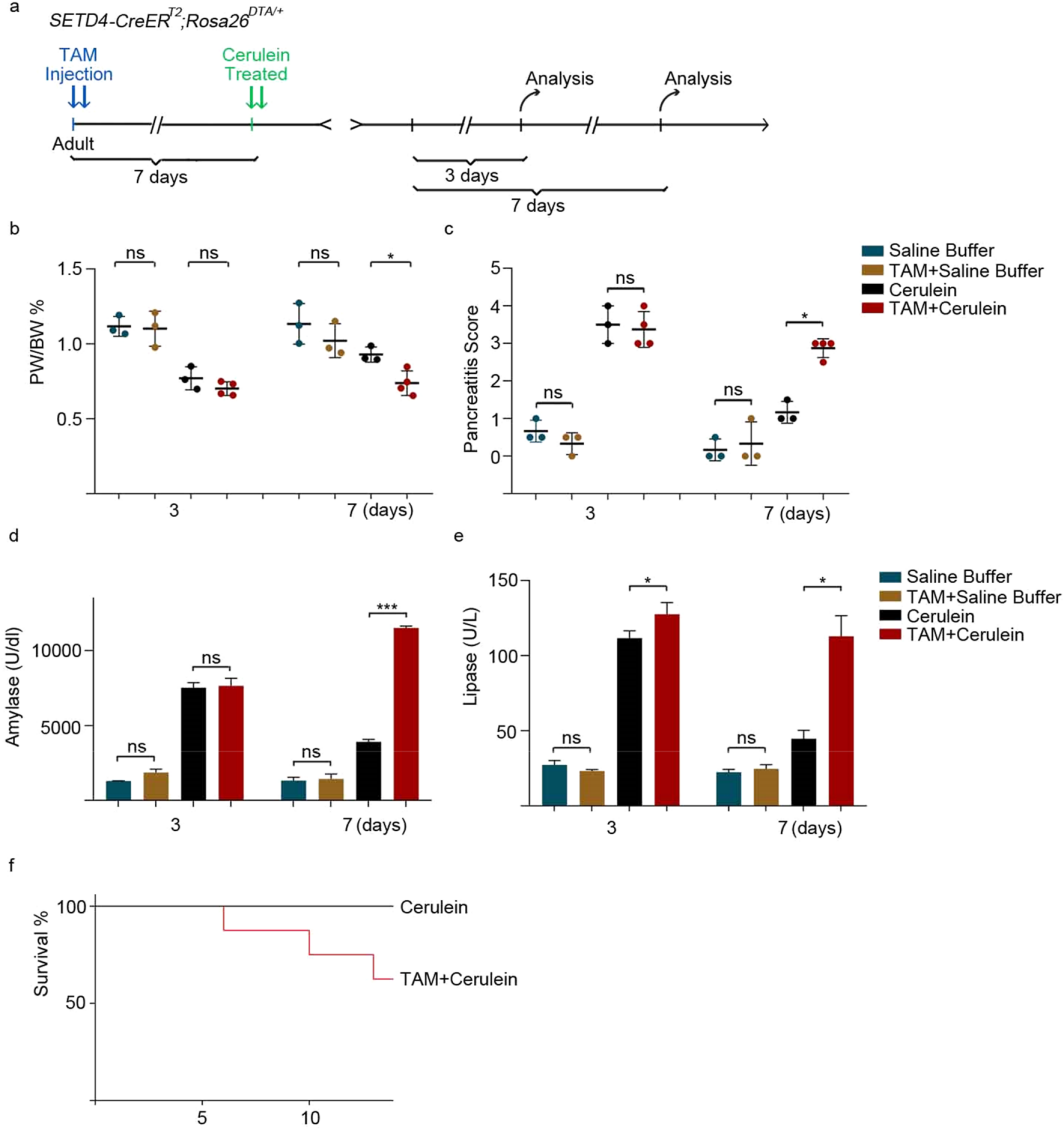
Effects of SETD4^+^ cells ablation after cerulein-induced injury in adult *SETD4-CreER*^*T2*^;*Rosa26*^*DTA/*+^ mice. (**a**) Experimental outline for *SETD4-CreER*^*T2*^;*Rosa26*^*DTA/*+^ mice. 8–10 week mice were injected with TAM twice over a 24-h period and subjected to an episode of experimental chronic pancreatitis to induced chronic pancreas damage. Mice were sacrificed at two time points: 3 or 7 days after Cerulein treatment. (**b**) Statistics for PW/BW ratio after 3 or 7 days treatments. PW: pancreatic weight, BW: body weight. (**c**) Pancreatitis score after 3 or 7 days treatments. (**d**) and (**e**) Analysis of serum levels of amylase **(d)** and lipase **(e)** after 3 or 7 days of treatments. (**f**) survival rates after SETD4^+^ cell ablation in creulein and TAM + Cerulein mice. Ctrl: Cerulein treatment (n = 3 mice), TAM + Cerulein: cerulein treatment after TAM-induction. (n = 4 mice). All data are represented as mean ± SD. *p < 0.05. *ns* not significant.

To validate this, *SETD4-CreER*^*T2*^;*Rosa26*^*DTA/*+^ and control mice were induced with Tamoxifen, After 7 days, they were then fasted and subjected to cerulein treatment. Histology, the ratio of pancreas to body weight, pancreatitis score, serum amylase and lipase were all analyzed after 3 and 7 days of treatment (Fig. 5a and S5a, S5b). Control animals (Cerulein) recovered within 7 days from pancreatic injury while the ablation of SETD4^+^ cells (TAM + Cerulein) led to the inability to regain pancreatic weight and a decreased pancreatitis score (Fig. 5b,c). In addition, the ablation of SETD4^+^ cells (TAM + Cerulein) failed to decrease in the serum levels of amylase and lipase after TAM treatment, in contrast to controls (Cerulein) (Fig. 5d,e). Tunel assay and analyses of histology showed that with the ablation of SETD4^+^ cells, mice had still failed to repair cerulein induced pancreatitis 7 days after cerulein treatment (Fig. S5c, S5d, S5e) and began to die after 6 days of cerulein treatment (Fig. 5f). And masson staining revealed a high fibrotic index both in *SETD4-CreER*^*T2*^;*Rosa26*^*mTmG/*+^ (control mice) and *SETD4-CreER*^*T2*^;*Rosa26*^*DTA/*+^ mice 3 days after induction of chronic pancreatitis. However, after 7 days of treatment, the control mice had returned to normal levels whereas the *SETD4-CreER*^*T2*^;*Rosa26*^*DTA/*+^ mice still had high fibrotic index (Fig. S5f, S5g) which further proved that these results were due to SETD4 loss. Over all, these results indicated that the ablation of SETD4^+^ quiescent cells had a strongly detrimental effect on pancreatic repair.

## Discussion

Controversy has long surrounded the idea of pancreatic exocrine or endocrine cell regeneration. Some investigators support the concept of pancreatic plasticity, that pancreatic exocrine cells can trans-differentiate to a progenitor-like cell in response to injury^2,3,5,7^. Others suggest the probable existence of pancreatic quiescent cells within the adult pancreas that are able to constantly replenish the cell pool required for homeostasis or for repair after injury^50,51^. In support of the second case, doublecortin-like kinase-1 (Dclk1) had been previously noted as a marker of a small population of pancreatic quiescent cells that participate in cerulein-induced injury repair, their loss seeming to have detrimental effect on cerulein-induced pancreatitis^33^. Our discovery of SETD4^+^ cells provides added confirmation of a population of quiescent pancreatic cells. However, differing from the previously noted Dclk1^+^ quiescent cells, we found that SETD4^+^ cells not only largely contribute to regeneration in cerulein-induced pancreatitis, but also contribute to pancreas development both in the embryonic and postnatal pancreas. In addition, in the exocrine pancreas, *Bmi1* is expressed in a subpopulation of acinar cells that show a differentiated phenotype which has also been reported to be able to maintain pancreatic organ homeostasis. The use of diphtheria toxin cell ablation (DTA)^52^ and cerulein-induced pancreatitis injury models demonstrated that the Bmi1-labeled, differentiated acinar cells undergo compensatory proliferation to maintain organ homeostasis^53^.

Previous studies have shown that Sox9, Pdx1 and Nkx6.1 define multipotent pancreatic progenitor cells in embryonic stages. In the adult, Sox9 is required for maintenance of the function of duct cells whereas Pdx1^+^ and Nkx6.1^+^ cells are required for the maintenance of glucose homeostasis. Therefore, Sox9 could be considered as the marker of embryonic pancreas or duct progenitor, whereas Pdx1 and Nkx6.1 are to be considered as markers of embryonic pancreatic progenitors or pancreatic endocrine cells. In this study, we have quantified the expression levels of these genes in FACS-sorted SETD4^+^ (GFP^+^) and SETD4^-^ (GFP^-^) cells by qPCR. Results showed that *Sox9*, *Pdx1* and *Nkx6.1* were more highly expressed in SETD4^+^ cells than SETD4^-^ cells. The characteristic of high expression level of these genes in SETD4^+^ cells indicated SETD4^+^ cells are distinct from other differentiated pancreatic cells. Our subsequent experiment indeed proved that SETD4^+^ cells exhibit higher degree of plasticity in the experiment beyond cerulein-induced injury and in organoid formation efficiency as observed in the in vitro self-organization into pancreatic organoids.

As an added note, SETD4^+^ cells and their progenies appeared in the E6 primitive layer which period is earlier than pancreas-specific development (Data not shown) which indicated SETD4 itself is not specific for the pancreas. SETD4^+^ cells have now been also identified in many tissues, such as the gut, brain and heart (Data not shown) which suggest that SETD4^+^ cells might also play important role in these tissues. Investigations, in our own laboratory, are in progress. In addition, in the conclusion of lineage tracing for SETD4^+^ cells in embryo and adult pancreas, we stated that SETD4^+^ cells could produce each pancreatic lineage which does not mean that a cell type of SETD4^+^ cells give all pancreatic lineages. Here, the SETD4^+^ cells in E6 primitive layer or E9 pancreas might have the ability to give rise to each pancreatic lineage. While, in adult pancreas, it’s possible that a cell type that gives rise to each lineage. Another possibility is that different types of SETD4^+^ cells produce specific pancreatic lineages. Such as, the SETD4^+^ cells located in acinar compartment only have the ability to produce the acinar cells.

In conclusion, we have previously reported an evolutionarily conserved mechanism in which SETD4 epigenetically controls cellular quiescence by facilitating heterochromatin formation via H4K20me3 catalysis. In addition, we demonstrated that H4K20me3 localized to the promoter regions and negatively correlated with the expression of the genes such as *MYC*, *WNT1*, *EEF1A1*, *IGF1*, *SMAD4*, and upregulated the expression, such as *TP53*, *BMP* and *BMI1* gene^44^. Here, we found that SETD4^+^ cells are in a nondividing state and lack of the cell proliferation markers of Ki67 and PCNA. We thus propose that the cellular quiescence in the pancreas is regulated by SETD4 according to an evolutionarily conserved mechanism. Our findings suggest that activated SETD4^+^ cells could be used as key targets in clinical treatment for a wide range of pancreatic diseases.

## Materials and methods

### Ethical approval statement

All experimental procedures followed the ARRIVE guidelines. All experimental protocols were also approved by Zhejiang University Animal Experiment Facility. We confirm that all experiments were performed in accordance with the relevant guidelines and regulations. Animal maintenance and experimentation were conducted in accordance with the ethical and practical guidelines of the Institutional Animal Care and Use Committee at Zhejiang University.

### Mouse strains, maintenance and genotyping

The *SETD4-CreER*^*T2*^ line was generated by homologoues (Fig. S1a), and the *SETD4-Cre* line was generated by CRISPR/Cas9 (Fig. S2b). The offspring of *SETD4-CreER*^*T2*^ line and *SETD4-Cre* were crossed with Rosa26-MTMG (no. 007676; from The Jackson Laboratory) and the offspring of *SETD4-CreER*^*T2*^ line was crossed with Rosa26-DTA (no. 010527; from The Jackson Laboratory). The *SETD4-Cre*, *SETD4-CreER*^*T2*^, were generated by Shanghai Model Organisms Center, Inc. All experimental mice were maintained on a C57BL/6; 129 background.

All mice were maintained in a pathogen-free environment and housed in clear shoebox cages in groups of four to six animals per cage with constant temperature and humidity and 12/12 h light/dark cycles. Studies were carried out in accordance with institutional guidelines. Activation of the CreER^T2^ was obtained by intraperitoneal injection for corn oil-dissolved Tamoxifen (20 mg/mL, Sigma T5648). For postnatal and adult lineage tracing experiments and injury induction, mice were given 160 mg/kg of Tamoxifen via oral gavage. Genotyping was performed by conventional PCR on genomic DNA isolated from mouse-tails using standard procedures according to the instruction of Quick Genotyping Assay Kit for Mouse Tail (Beyotime, China D7283S).

### Tissue preparation

Mice were anaesthetized with 50 mg/kg pentobarbital sodium by intraperitoneal injection. The pancreas was collected and then fixed in 4% paraformaldehyde (PFA, Sigma P6148) at 4 °C, and washed in PBS extensively. For frozen sections, samples were dehydrated in 30% sucrose overnight at 4 °C, and embedded in optimal cutting temperature compound (Sakura #4583). For paraffin embedded sections, samples were dehydrated in gradient alcohol and then mounted in paraffin.

### Acinar, duct and islet cells isolation

#### Acinar cell isolation

The pancreas was extracted, and adipose tissue was removed. Four solutions were prepared, including D solution (1 mg/mL Collagenase Type CLS IV supplemented with 0.25% BSA [Sigma-Aldrich]), R solution (1% BSA dissolved in PBS), C solution (4% BSA in PBS), and I solution (0.1% BSA in PBS). The tissue was chopped into small pieces and incubated in 10 mL of D solution at 37 °C for 30 min. The digestion product was filtered through a 70-mm cell strainer (islets of Langerhans were thereby removed). Ten milliliters of R solution were pipetted on the cell strainer. A quarter of the filtered cell suspension was gently transferred on top of 6 mL of C solution to achieve layer separation of the liquids. Acini were spun down at 50×*g* for 2 min and washed with C solution and I solution successively. Purified acini were treated with 2 mL of Accutase (Sigma-Aldrich) for 5 min to acquire acinar cell suspension, and then single acinar cells were acquired.

### Duct cells isolation

The mouse was sacrificed according to approved institutional guidelines. The pancreas was then extracted, and adipose tissue was removed. After that, the pancreas was harvested and transferred to a dish containing cold DMEM/F-12. The pancreas was cut up into small (~ 3 mm) pieces and then transferred into a 50 ml tube. Pancreatic ductal cells were isolated by Tissue Dissociation Cocktail (Collagenase type IV, (Sigma C5138), Dispase (Stem Cell Technologies #07909), DNase 1 solution (Stem Cell Technologies #07900), DMEM/F12 with 15 mM HEPES (Gibco #11330032). The specific isolation and digestion method was to put the pancreas pieces containing the Tissue Dissociation Cocktail Buffer in a 37 °C water bath for 20 min and then collect the supernatant. The digestion steps were repeated 6–10 times. The pancreatic ducts will be released into the supernatant with each successive digestion cycle. The collected supernatant was passed through a 70 µm cell strainer and the flow­through was discarded. The strainer was reversed onto a pre-wetted 50 ml conical tube. The cold DMEM/F-12 was added to the reversed strainer to wash tissue fragments and ducts. The ducts were then collected by centrifugation at 290×*g* for 5 min. Finally, the pancreas ducts were dissociated into single duct cells by addition of trypsin (1 mg/ml, Sigma) and DNase 1 (0.4 mg/ml).

### Islet cell isolation

All pancreases were collected and placed in the digestion tubes containing Collagenase Type V (0.8 mg/ml) into a 37 °C water bath for 30 min. During this period, the tissue block was blown every ten minutes. The digestion tubes were then filled with cold wash buffer to 50 ml and mixed by inverting the tubes 5 times. The tubes were centrifuged at 97×*g* at 4 °C for 1 min and the supernatant was poured off. A30-mesh tissue sieve was placed over a sterile 250 ml beaker and the tissue suspension was poured onto the sieve. The digestion tube was then rinsed with an additional 20 ml of wash buffer and the digestive tissue was poured onto the mesh. The filtered material was then poured into two fresh 50 ml tubes and pelleted by spinning at 97×*g* for 1 min at 4 °C. The supernatant was decanted, and the tubes were inverted to drain excess buffer. 20 ml of cold polysucrose/sodium diatrizoate solution (1.119 g/ml density) was added to the pelleted pancreatic tissue. The contents of each tube were homogenously re-suspended by being gently pipetted up and down five times. Then the polysucrose/sodium diatrizoate solution was gently overlayed with 10 ml HBSS via being slowly added along the tube wall to maintain a sharp liquid interface. The digested pancreas was centrifuged at 560×*g* for 15 min with slow acceleration and no brake. The islet layer was collected from the interface using a 10 ml pipette and placed in fresh 50 ml tubes. 40 ml of wash buffer was added to each 50 ml tube and this was spun for 1 min at 140×*g* at 4 °C. The supernatant was poured off and the pooled islets in 50 ml of wash buffer were re-suspended by inverting the tubes several times. The tubes were centrifuged for 1 min at 97×*g* at 4 °C to collect islets in a pellet. The islets were brought into the tissue culture hood and the supernatant was aspirated. The islets were then transferred from each 50 ml tube into a well of a six-well tissue culture dish. The 6-well dish was swirled to collect islets into the middle of the well and islet quality and purity were observed under a microscope. The islets were manually collected using a 1 ml pipette and transferred into the 6-well dish and then dissociated into single cells by addition of Trypsin (1 mg/ml, Sigma) and DNase 1 (0.4 mg/ml).

### Quantitative real-time PCR

Cells in acinar, duct and islet compartments were sorted from adult *SETD4-CreER*^*T2*^;*Rosa26*^*mTmG/*+^ mice 24 h after TAM induction and were processed using ReliaPrep RNA Cell Mini prep System (Z6010, Promega, Milan, Italy). Reverse transcription (RT) was conducted using a High-Capacity cDNA Reverse Transcription Kit (4368814, Applied Biosystems). Quantitative Real-Time PCR reactions were performed on the Bio-Rad MiniOpti-con system using SYBR Premix Ex. Taq (TaKaRa Bio; RR420A). The relative amounts of mRNAs were analyzed using the comparative CT method, as described previously^54^.

### Flow cytometry and pancreas organoid culture

8–10 weeks old *SETD4-CreER*^*T2*^;*Rosa26*^*mTmG/*+^ mice were injected with tamoxifen. After 24 h, pancreatic ducts were isolated using by Tissue Dissociation Cocktail (Collagenase type IV, (Sigma C5138), Dispase (Stem Cell Technologies #07909), DNase 1 solution (Stem Cell Technologies #07900), DMEM/F12 with 15 mM HEPES (Gibco #11330032) and then dissociated into single cells by the addition of trypsin (1 mg/ml, Sigma) and DNase 1 (0.4 mg/ml). The cell suspension was then filtered through a 70-mm cell strainer and stained with CD45 for 30 min at 4 °C and washed with 0.1% bovine serum albumin (BSA) in PBS. The cells without any antibody served as unstained controls for setting gates. 7-AAD (BD Biosciences #51-68981E) was added to the stained tube 10 min before acquiring for evaluating viability. The tdTomato (mT) served as the PE marker to gate out the GFP^+^ cells. All the doublets were removed by FSC-A vs FSC-H scatter. VSELs were quantitated as percentage total of five lakh events acquired and results were analyzed on FACS Diva software (BD FACSDiva 8.0.1). Sorted GFP^+^-CD45^–^7-AAD^-^ duct cells were mixed with growth factor-reduced Matrigel (Corning #356231) to a final density of 100 cells/well and seeded in 96-well plates. After gelation of the Matrigel, pancreatic organoid growth medium (Stem Cell Technologies #06040) was added in each well and replenished every 3 days. Organoids usually formed on the third day after seeding cells in culture and were passaged for the first time after 7 to 10 days. GFP^–^CD45^–^7-AAD^-^ duct cells were cultured in the same way.

### Experiment of inducing chronic pancreatitis

8–10 weeks old *SETD4-CreER*^*T2*^;*Rosa26*^*mTmG/*+^ mice/*SETD4-CreER*^*T2*^;*Rosa26*^*DTA/*+^ mice were randomly divided into groups of 4–6 animals, and then injected with tamoxifen twice over 24 h. After they were fasted for 12 h, they were then subjected to an episode of experimental chronic pancreatitis to induced pancreatic damage. Mice received 2 intraperitoneal injections of 50 μg/kg cerulein (Meilun MB2573) at six hourly intervals for a day, two days a week for 2 weeks. Control mice from all genotypes received comparable injections of 0.9% saline buffer. Mice were sacrificed at several time points: 3, 7 or 14 days after injection with cerulein. 5-ethynyl-2’-deoxyuridine (EdU) solution was injected in mice 2 h before sample collected. Body weight was examined in each time point.

### TUNEL assay

In the pancreatic tissue, apoptosis was detected by using of YF488 TUNEL Assay Apoptosis Detection Kit (US Everbright T6013), according to the manufacturer's instructions. Briefly, the tissue sections were incubated with TUNEL reaction mixture at 37˚C for 1 h. TUNEL-positive cells displayed brilliant green fluorescence. For each test, negative controls were included. 8–10 high-power fields were randomly selected and TUNEL-positive cells were counted in each field.

### Pancreatitis score

Signs of pancreatitis were scored as described before^48^ and ten high power fields were scored per mouse for inflammation, vacuoles, acinar necrosis and edema. The mean for each item was aggregated to yield the overall histological pancreatitis score.

### EdU administration

To determine the presence of proliferating cells, mice were injected (i.p.) with 5-ethynyl-2-deoxyuridine (EdU, 10 mg/kg). After 2 h, pancreases were isolated. Section stained using Click-iT EdU Alexa Fluor 647 Imaging kit (Life Technologies) according to the manufacturer's instructions.

### Western blotting

Total cellular proteins were extracted by RIPA lysis buffer (Beyotime P0013B) containing protease inhibitor cocktail (MedChemEx-191 press; HY-K0010); Tissue total proteins were prepared using the Trizol Extraction Reagent (Invitrogen #15596018) according to manufacturer instructions. Protein concentration was resolved by SDS-PAGE and transferred to PVDF membranes (Bio-RAD #1620177). The filters were blocked in 1% BSA for 1 h at room temperature and then incubated with primary antibody overnight at 4 °C. The filters were washed 3 times in TBS and incubated in secondary antibodies overnight. Membrane-bound immune complexes were detected by ultra-sensitive enhanced chemiluminescence system (Bio-RAD #102031152) on Amersham Imager 600 (GE). Primary antibodies were used as below: mouse anti-SETD4 (1:50, Santa Cruz Biotechnology sc-514060); Full antibody details are provided in the supplemental information antibodies list. Quantification was performed by densitometric analysis using ImageJ 64 software.

### Hematoxylin–eosin staining, Masson staining and immunofluorescence

The pancreas from 8 to 10 week mice were removed and fixed in 4% paraformaldehyde overnight, washed in PBS overnight, dehydrated in gradient alcohols, and embedded in paraffin. 8 µm sections were stained with hematoxylin–eosin and photographed using a Zeiss Axioplan light microscope.

For masson staining, adult *SETD4-CreER*^*T2*^;*Rosa26*^*mTmG/*+^ and *SETD4-CreER*^*T2*^;*Rosa26*^*DTA/*+^ mice were injected with cerulein after TAM-induction. 8 to 10-week mice were randomly divided into groups of 4–6 animals and injected with TAM twice over a 24-h period. After7 days, they were then fasted for 12 h and subjected to an episode of experimental chronic pancreatitis to induced chronic pancreatitis damage. Control group received comparable injections of 0.9% saline buffer. Mice were sacrificed at two time points: 3 or 7 days after treatment. Pancreas sections (6 μm thickness) were prepared using a microtome, and placed on glass slides. Staining was conducted using Masson (Masson Stain Kit, YESEN) according to the manufacturer’s instruction. Masson’s stained sections were evaluated to quantify the amount of fibrosis by calculating the area occupied by blue-stained collagen using the ImageJ program.

Dissected whole embryos (E9.0) and pancreases (E15.5, P0 and P56) were fixed in 4% paraformaldehyde in PBS at 4 °C for between 45 min (E9.0 embryos) and 3 h (E15.5, P0 and P56 pancreata) dependent upon tissue volume. They were then dehydrated in 30% sucrose in PBS overnight at 4 °C, equilibrated in Tissue-Tek O.C.T. for 1 h at room temperature (RT) were then cryoembedded in O.C.T. on dry ice slabs. 12–16 µm PFA-fixed frozen sections were prepared for immunofluorescence. For immunofluorescence, slides were fixed with 2% PFA and washed with cold PBS. Antigen retrieval for frozen section was conducted via incubation in antigen retrieval solution (Sangon Biotech E673009) for 5 min and then rinsing and blocking for 1 h with 3% normal donkey serum with 0.5% Triton X-100. Primary antibodies and fluorophore-conjugated secondary antibodies were diluted in 3% normal donkey serum with 0.5% Triton X-100. Primary antibodies were incubated over night at 4 °C and secondary antibodies were incubated for 2 h at RT. Slides were mounted in Antifade Mounting Medium with DAPI (Beyotime P0131).

For EdU detection, Fluorescent EdU detection was done using the Baseclick EdU Proliferation Detection Kit (Sigma BCK-EDU647) according to the manufacturer’s instructions. Primary antibodies were used as below: chicken anti-GFP (1:1000, Abcam ab13970); Full antibody details are provided in the supplemental information for Oligonucleotides.

### Measurements of serum amylase and lipase

Chronic pancreatitis was induced in 8–10 weeks old *SETD4-CreER*^*T2*^;*Rosa26*^*DTA/*+^ mice as described above. The experiment was divided into four group, including Cerulein and TAM + Cerulein treatment. The serum activities of amylase and lipase were measured by enzyme dynamics using commercial kits according to the manufacturer’s protocols (Sangon Biotech D799323-0050 and Sangon Biotech D799801-0050).

### Quantification and statistical analysis

#### Quantification of baseline recombination in *SETD4-CreER*^*T2*^;*Rosa26*^*mTmG/*+^ mice

The pancreases were trimmed of all nonpancreatic tissue, weighed, fixed, and cryoprotected in 30% sucrose overnight before freezing in a way to allow longitudinal sections from tail to head of the pancreas to be obtained. Sections were taken at 100-μm intervals from whole pancreas.

Recombination was assessed 24 h after treatment with tamoxifen and with 80–100 high power fields being randomly selected. Recombinant cells were counted manually and divided into acinar, duct and islet cells based on their respective morphology. To quantify the co-localization of SETD4 expression with other progenitor and mature marker expression, double SETD4^+^/each marker (Sox9, Pdx1, Nkx6.1, amylase, CK19, insulin, glucagon, somatostatin and ghrelin) positive cells were counted (n = 4 mice).

#### Quantification of recombination GFP^+^ cells in lineage tracing

In lineage tracing studies, fluorescent pictures were taken in both the green and the blue channel to visualize recombination and DAPI^+^ nuclei, respectively. Recombinant cells were counted manually and divided into acinar, ductal and islet cells based on their respective morphology (n ≥ 3 mice). To identify the progeny of SETD4^+^ cells, GFP-labeled amylase^+^, CK19^+^, glucagon^+^, insulin^+^, somatostatin^+^ and ghrelin^+^ cells on a section were traced, respectively. To calculate the proportion of clones, we randomly selected 50–70 recombinant acinar cells, 30–50 recombinant duct cells and 20–30 recombinant islet cells for each mouse based on their respective morphology and grouped them as either single cell (1), doublets (2), or clones of three or more cells (≥ 3). The percentage of each type of clones was calculated according to the total clones of each time point (n = 4 mice).

#### Quantification of recombination in *SETD4-CreER*^*T2*^;*Rosa26*^*mTmG/*+^ mice after cerulein induced injury

Recombination was assessed at days 3, 7 or14 after cerulein treatment. Treated saline buffer was used as a control and was analyzed in parallel. To assess acinar, duct and islet cell neogenesis, 30–50 high power fields for each mouse were randomly selected. Recombinant cells were counted manually and divided into acinar, ductal and islet cells based on their respective morphology (Control: n = 3 mice, Cerulein treatment: n = 4 mice).

#### Quantification of proliferated cells in *SETD4-CreER*^*T2*^;*Rosa26*^*mTmG/*+^ mice after cerulein induced injury

Immunofluorescence for EdU and Ki67 was performed at the day after cerulein treatment. Acinar cells were analyzed based on their respective morphology. 30–50 acinar cells for each mouse were randomly selected and EdU and Ki67 positivity was assessed in recombinant GFP^+^ cells, respectively (Control: n = 3 mice, Cerulein treatment: n = 4 mice). Mice treated with saline buffers were analyzed as controls in parallel.

#### Image processing, data analysis and statistical analysis

All non-confocal images of stained sections were acquired using a fluorescent microscope (Leica, Germany), Confocal images were acquired using an Olympus confocal laser (Olympus, FV3000, Japan). Statistical testing and was conducted using GraphPad Prism software with the appropriate test for each experiment. All data are shown as means with SD. All statistical analyses were performed in GraphPad Prism 8.0 software. For quantitative data analysis, the Student’s *t* test was performed for comparisons of 2 groups. Multiple comparisons test was conducted by two-way/three-way ANOVA with Bonferroni's correction. Statistical significance was depicted as follows: *p < 0.05, **p < 0.01 and ***p < 0.001, ns: not significant.

## Supplementary Information

Supplementary Information.
