# Supplementary material for: SETD4-expressing cells contribute to pancreatic development and response to cerulein induced pancreatitis injury
Source: Sci Rep. 2021 Jun 15;11:12614. doi: 10.1038/s41598-021-92075-5 (PMC8206148; doi:10.1038/s41598-021-92075-5)
Supplement: Supplementary file 1 — Supplementary Information. [file 41598_2021_92075_MOESM1_ESM.pdf]

Supplemental information

SETD4-expressing Cells Contribute to Pancreatic Development and Response to Cerulein Induced Pancreatitis Injury

Authors:

Jin-Ze Tian[1], Sheng Xing[1], Jing-Yi Feng[1], Shu-Hua Yang[1], Yan-Fu Ding[1], Xue-Ting Huang[1], Jin-Shu Yang[1], Wei-Jun Yang[1,2]*

Affiliations:

[1]MOE Laboratory of Biosystem Homeostasis and Protection, College of Life, Sciences, Zhejiang University, Hangzhou, 310058, China.

[2]Laboratory for Marine Biology and Biotechnology, Qingdao National Laboratory for Marine Science and Technology, Qingdao 266000, China

***Correspondence: w_jyang@zju.edu.cn**

Supplemental information includes one table and five figures.

Table 1 Key resources table

Regent or resource	Source	Identifier
**Antibodies**		
Mouse anti-SETD4 (1:50)	Santa Cruz Biotechnology	sc-514060
Rabbit anti-Nkx6.1 (1:100)	Abcam	ab221549
Rabbit anti-Pdx1(1:100)	Abcam	ab47262
Goat anti-Pdx1 (1:10000)	Abcam	ab47383
Rabbit anti-Sox9 (1:200)	Millipore	AB5535
Rabbit anti-Cytokeratin 19 (1:100)	Abcam ab52625	ab52625
Mouse anti-Amylase (1:200)	Santa Cruz	sc-46657
Rat anti-Insulin (1:100)	R&D Systems	MAB1417
Rabbit anti-Glucagon (1:100)	Abcam	ab92517
Mouse anti-Somatostatin (1:200)	Santa Cruz	sc-74556
Mouse anti-ghrelin (1:200)	Santa Cruz	sc-517596
Rabbit anti-Ki67 (1:100)	Abcam	ab16667
Chicken anti-GFP (1:1000)	Abcam	ab13970
Rabbit anti-PCNA (1:100)	Abcam	ab92552
Rat PE-Cy7 anti-CD45 (1:100)	BD Pharmingen	561868
**Chemicals, Peptides and Recombinant Proteins**		
Paraformaldehyde (PFA)	Sigma	P6148
Tamoxifen (TAM)	Sigma-Aldrich (Merck)	Cat#T5648
Cerulein	Meilun	MB2573
Collagenase type IV	Sigma	C5138
**Experimental Models: Organisms/Strains**		
Mouse: SETD4-CreER^{T2}	This paper	N/A
Mouse: SETD4-Cre	This paper	N/A
Mouse: Rosa26-MTMG	The Jackson Laboratory	no. 007676
Mouse: Rosa26-DTA	The Jackson Laboratory	no. 010527
**Oligonucleotides**		
Primer: mTmG-P1 5'-CTCTGCTGCCTCCTGGCTTCT-3'	This paper	N/A
Primer: mTmG-P2 5'-CGAGGCGGATCACAAGCAATA-3'	This paper	N/A
Primer: mTmG-P3 5'-TCAATGGGCGGGGGTCGTT-3'	This paper	N/A

Primer: DTA-wildtype F 5'-AAAGTCGCTCTGAGTTGTTAT-3'	This paper	N/A
Primer: DTA-wildtype R 5'-GGAGCGGGAGAAATGGATATG-3'	This paper	N/A
Primer: DTA-mutant F 5'-AAAGTCGCTCTGAGTTGTTAT-3'	This paper	N/A
Primer: DTA-mutant R 5'-GCGAAGAGTTTGTCCTCAACC-3'	This paper	N/A
Primer: Foxg1-Cre-A 5'-CACCCTGTTACGTATAGCCG-3'	This paper	N/A
Primer: Foxg1-Cre-B 5'-GAGTCATCCTTAGCGCCGTA-3'	This paper	N/A
Primer: Sox9 F 5'-GAGCCGGATCTGAAGAGGGA-3'	This paper	RRID:20682
Primer: Sox9 R 5'-GCTTGACGTGTGGCTTGTTC-3'	This paper	RRID:20682
Primer: Dclk1 F 5'-CTGGGTTAATGATGATGGTCTCC-3'	This paper	RRID:13175
Primer: Dclk1 R 5'-TCCTGGTTGTTGGTAGTAGTCC-3'	This paper	RRID:13175
Primer: Nkx6.1 F 5'-CTGCACAGTATGGCCGAGATG-3'	This paper	RRID:18096
Primer: Nkx6.1 R 5'-CCGGGTTATGTGAGCCCAA-3'	This paper	RRID:18096
Primer: pdx1 F 5'-CCCCAGTTTACAAGCTCGCT-3'	This paper	RRID:18609
Primer: pdx1 R 5'-CTCGGTTCCATTCGGGAAAGG-3'	This paper	RRID:18609
Primer: SETD4 F 5'-GGTAAAAGCGGCGTTTAACGA-3'	This paper	RRID:224440
Primer: SETD4 R 5'-GGATTGCGGACGGAGACAAA-3'	This paper	RRID:224440
**Software and Algorithms**		
Fiji (ImageJ v. 2.0.0)	NIH	RRID:SCR_002285
Microsoft Excel	Microsoft	RRID:SCR_016137
Adobe Photoshop (CS6)	Adobe	RRID:SCR_014199
GraphPad Prism version 8.0	GraphPad	RRID:SCR_002798
ZEN	Zeiss	RRID:SCR_013672
FV31S-SW	FV3000	N/A
FACS Diva software 8.0.1	BD	N/A
**Other**		
Tissue-Tek O.C.T.Compound	Sakura	Cat#4583
Quick Genotyping Assay Kit for Mouse Tail	Beyotime	D7283S
Dispase	Stem Cell Technologies	Cat#07909

DNase 1 solution	Stem Cell Technologies	Cat#07900
DMEM/F12 with 15 mM HEPES	Gibco	Cat#11330032
7-AAD	BD Biosciences	Cat#51-68981E
Growth factor-reduced Matrigel	Corning	Cat#356231
PancreaCult organoid growth medium	Stem Cell Technologies	Cat#06040
YF 488 TUNEL Assay Apoptosis Detection Kit	US Everbright	T6013
RIPA lysis buffer	Beyotime	P0013B
Protease inhibitor cocktail	MedChemEx-191 press	HY-K0010
Trizol Extraction Reagent	Invitrogen	Cat#15596018
PVDF membranes	Bio-RAD	Cat#1620177
Ultra-sensitive enhanced chemiluminescence system	Bio-RAD	Cat#102031152
Frozen section antigen retrieval solution	Sangon Biotech	E673009
DAPI	Beyotime	P0131
Baseclick EdU Proliferation Detection Kit	Sigma	BCK-EDU647
ReliaPrep RNA Cell Miniprep System	Promega	Z6010
α-Amylase Acitivity Assay Kit	Sangon Biotech	D799323-0050
Lipase (LPS) Acitivity Assay Kit	Sangon Biotech	D799801-0050
Masson stain Kit	YESEN	Cat#60632ES58

Figure S1

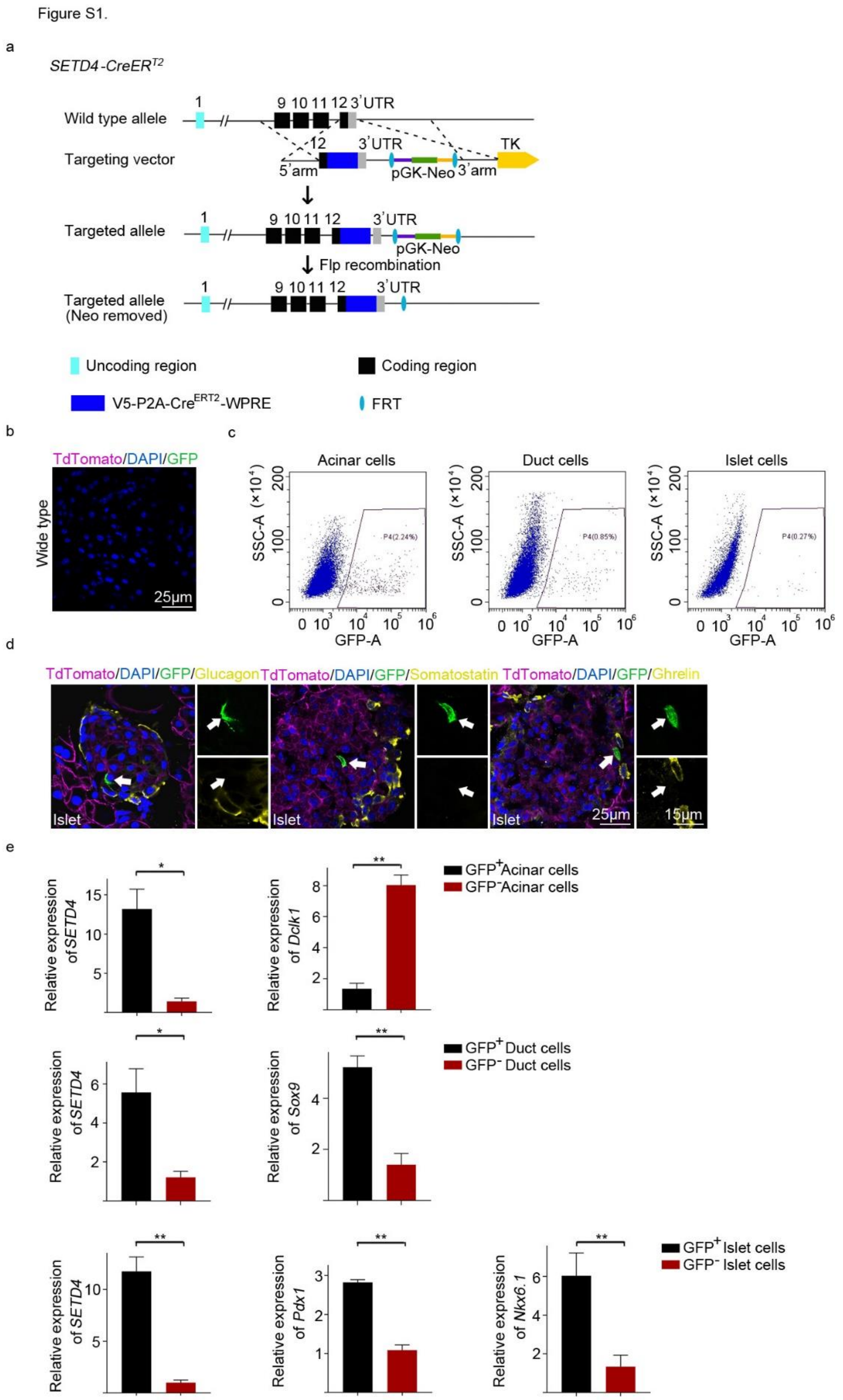

Figure S1.
a
SETD4-CreERT2
Wild type allele
Targeting vector
Targeted allele
Flp recombination
Targeted allele (Neo removed)
9 10 11 12 3'UTR
5'arm
3'arm
pGK-Neo
TK
Uncoding region
Coding region
V5-P2A-CreERT2-WPRE
FRT
b
TdTomato/DAPI/GFP
Wide type
25μm
c
Acinar cells
Duct cells
Islet cells
SSC-A (×10⁴)
GFP-A
P4(2.24%)
P4(0.85%)
P4(0.27%)
d
TdTomato/DAPI/GFP/Glucagon
TdTomato/DAPI/GFP/Somatostatin
TdTomato/DAPI/GFP/Ghrelin
Islet
25μm
15μm
e
Relative expression of SETD4
Relative expression of Dclk1
Relative expression of Sox9
Relative expression of Pdx1
Relative expression of Nkx6.1
GFP+ Acinar cells
GFP- Acinar cells
GFP+ Duct cells
GFP- Duct cells
GFP+ Islet cells
GFP- Islet cells

Figure S1: Generation of *SETD4-CreER*^{T2} lines and characterization of SETD4^{+} cells in *SETD4-CreER*^{T2}*;Rosa26*$^{mTmG/+}$ adult mice after 24 hours TAM-induction.

(**a**) Schematic diagram of the gene targeting strategy for generation of the *SETD4-CreER*^{T2} knock-in line by homologous recombination. A V5-P2A-Cre^{ERT2}-WPRE-polyA cassette was fused behind Exon 12 of *SETD44* in ES cells by electroporation. G418 and Ganc selection of resistant clones were performed to screen for homologous recombination. Targeted ES clones were microinjected into C57BL/6J eight-cell stage embryos and transferred into pseudo-pregnant C57BL/6J females. The obtained chimeras were crossed with Flp mice to generate neo-free *SETD4-CreER*^{T2} mice.

(**b**) Representative DAPI staining in wide-type mice.

(**c**) Representative flow cytometric scatter plots. Isolation by flow cytometry analysis of GFP^{+} cells from each compartment from *SETD4-CreER*^{T2};*Rosa26*$^{mTmG/+}$ mice after 24 hours TAM induction. Dissociated cells were gated as CD45 negative cells, GFP^{+}-CD45^{-} cells were then counted.

(**d**) Representative immunofluorescence for glucagon, somatostatin, and ghrelin of GFP^{+} cells from *SETD4-CreER*^{T2};*Rosa26*$^{mTmG/+}$ mice after 24 hours TAM induction.

(**e**) Quantitative Real-Time PCR analysis of endogenous *SETD4* and *Dclk1* in GFP^{+} and GFP^{-} acinar cells, *SETD4 and Sox9* expression in GFP^{+} and GFP^{-} duct cells, *Pdx1* and *Nkx6.1* expression in GFP^{+} and GFP^{-} islet cells from *SETD4-CreER*^{T2}*;Rosa26*$^{mTmG/+}$ mice after 24 hours TAM induction were sorted by fluorescence activated cell sorting (FACS).

All data are represented as mean ± SD. *$p < 0.05$, **$p < 0.01$. Nuclei were stained with DAPI.

FigureS2

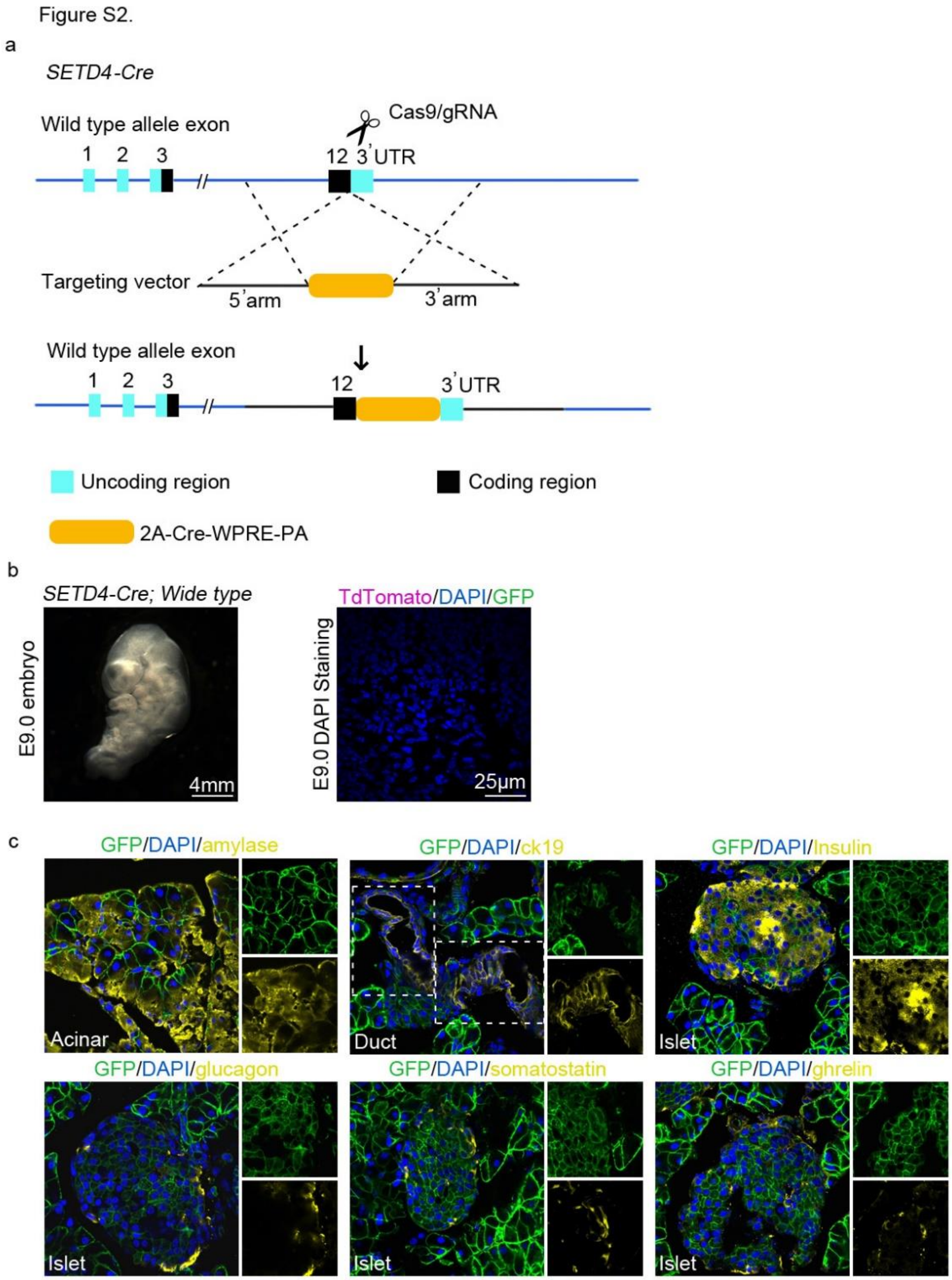

Figure S2.
a
SETD4-Cre
Wild type allele exon
Cas9/gRNA
1 2 3
12 3'UTR
Targeting vector
5'arm
3'arm
Wild type allele exon
1 2 3
12
3'UTR
Uncoding region
Coding region
2A-Cre-WPRE-PA
b
SETD4-Cre; Wide type
E9.0 embryo
4mm
TdTomato/DAPI/GFP
E9.0 DAPI Staining
25μm
c
GFP/DAPI/amylase
Acinar
GFP/DAPI/ck19
Duct
GFP/DAPI/Insulin
Islet
GFP/DAPI/glucagon
Islet
GFP/DAPI/somatostatin
Islet
GFP/DAPI/ghrelin
Islet

Figure S2: Generation of *SETD4-Cre* knock-in lines and identification the descendants of SETD4^{+} quiescent cells in P0 *SETD4-Cre*;*Rosa26*$^{mTmG/+}$ mice.

(**a**) Schematic diagram of the gene targeting strategy to generate the *SETD4-Cre* knock-in line via CRISPR/Cas9. A vector was generated in which a T2A-Cre-WPRE-PA cassette was fused with the 3'-end SETD4 coding region. Cas9 mRNA, gRNA, and the donor vectors were microinjected into fertilized ova of C57BL/6J mice to obtain F0 generation mice. Positive F0 generation mice were identified by PCR and DNA sequencing and then crossed with C57BL/6J mice to obtain positive F1 generation mice.

(**b**) The E9.0 whole embryo which generated from *SETD4-Cre* mice crossed with wide-type mice and DAPI staining for corresponding sections.

(**c**) Representative immunofluorescence for amylase, CK19, insulin, glucagon, somatostatin and ghrelin in the P0 pancreas from *SETD4-Cre*;*Rosa26*$^{mTmG/+}$ mice. The dotted box represents the duct compartment.

Figure S3

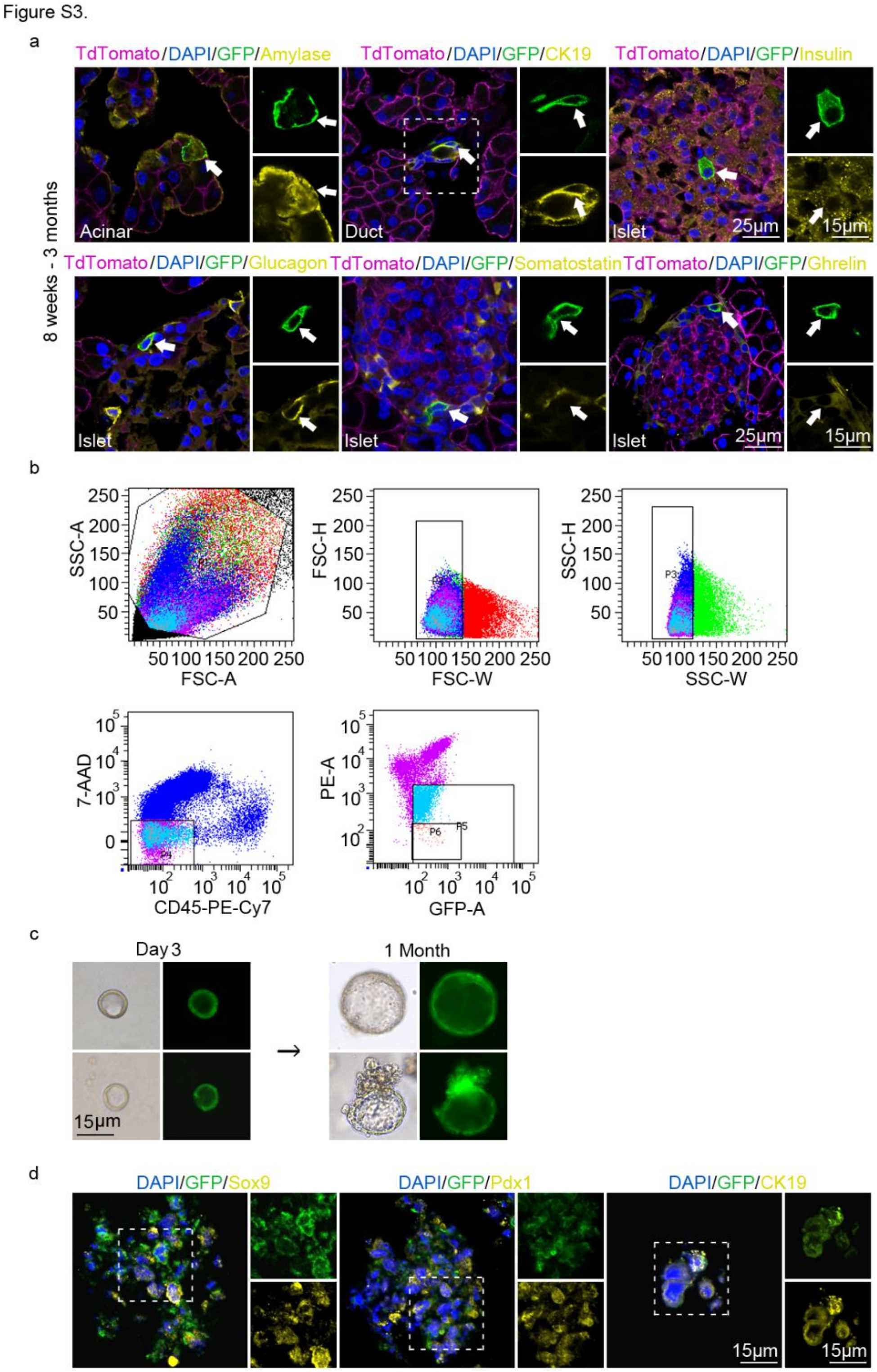

Figure S3.
a
TdTomato/DAPI/GFP/Amylase
TdTomato/DAPI/GFP/CK19
TdTomato/DAPI/GFP/Insulin
Acinar
Duct
Islet
25μm
15μm
8 weeks - 3 months
TdTomato/DAPI/GFP/Glucagon
TdTomato/DAPI/GFP/Somatostatin
TdTomato/DAPI/GFP/Ghrelin
Islet
Islet
Islet
25μm
15μm
b
SSC-A
FSC-A
FSC-H
FSC-W
SSC-H
SSC-W
P3
7-AAD
CD45-PE-Cy7
PE-A
GFP-A
P5
P6
c
Day 3
1 Month
15μm
d
DAPI/GFP/Sox9
DAPI/GFP/Pdx1
DAPI/GFP/CK19
15μm
15μm

Figure S3: Lineage tracing of SETD4$^+$ cells in *SETD4-CreER^{T2};Rosa26$^{mTmG/+}$* mice and cell-organized organoids of SETD4$^+$ cells isolated from the ducts.

(**a**) Representative immunofluorescence for amylase, CK19, insulin, glucagon, somatostatin and ghrelin in the adult pancreas (8 weeks) after 1 month of TAM-induction.

(**b**) Isolation by fluorescence activated cell sorting of GFP recombinant SETD4$^+$ cells from the ducts of 5 individual *SETD4-CreER^{T2};Rosa26$^{mTmG/+}$* mice after 24 hours TAM-induction. Results shown as gated on forward scatter (FSC) or side scatter (SSC). Dissociated duct cells were gated as CD45 negative cells, and 7-AAD was used to exclude dead cells. GFP$^+$-CD45$^-$-7-AAD$^-$ population (P6) were sorted.

(**c**) Representative images of pancreatic organoids of SETD4$^+$ cells (green) after growth in 3-Dimentional Matrigel-based culture for 3 days or 1 month after culturing. Scale bar for all each image, 15 μm.

(**d**) Representative immunofluorescence for Sox9, Pdx1 and CK19 in recombinant GFP$^+$ organoids. Dotted boxes represent magnification areas of immunofluorescent images. Nuclei were stained with DAPI. n = 3 mice.

Figure S4

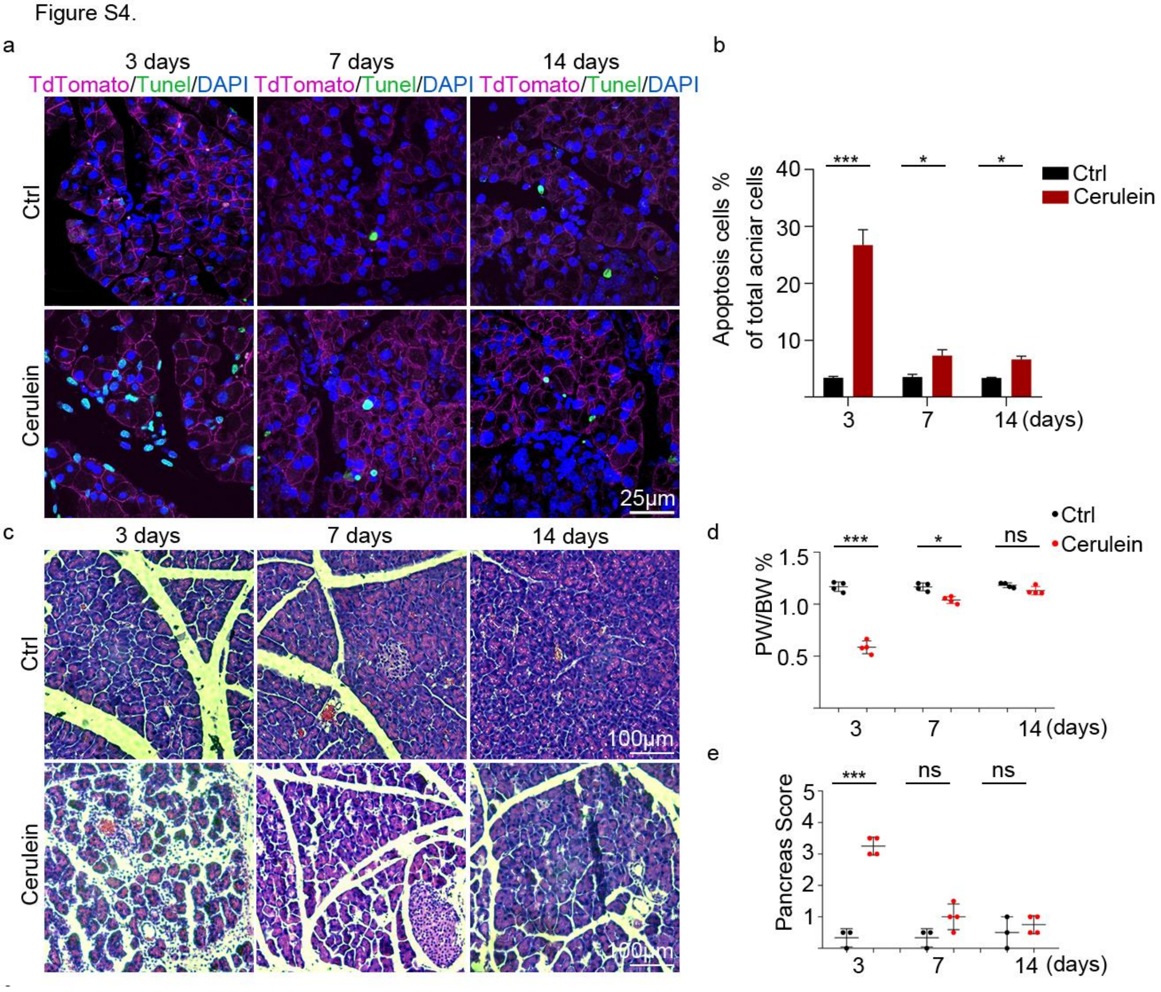
Figure S4.
a
3 days
7 days
14 days
TdTomato/Tunel/DAPI
TdTomato/Tunel/DAPI
TdTomato/Tunel/DAPI
Ctrl
Cerulein
25μm
b
Apoptosis cells % of total acniar cells
40
30
20
10
***
*
*
Ctrl
Cerulein
3
7
14 (days)
c
3 days
7 days
14 days
Ctrl
Cerulein
100μm
100μm
d
PW/BW %
1.5
1.0
0.5
***
*
ns
•Ctrl
•Cerulein
3
7
14 (days)
e
Pancreas Score
5
4
3
2
1
***
ns
ns
3
7
14 (days)

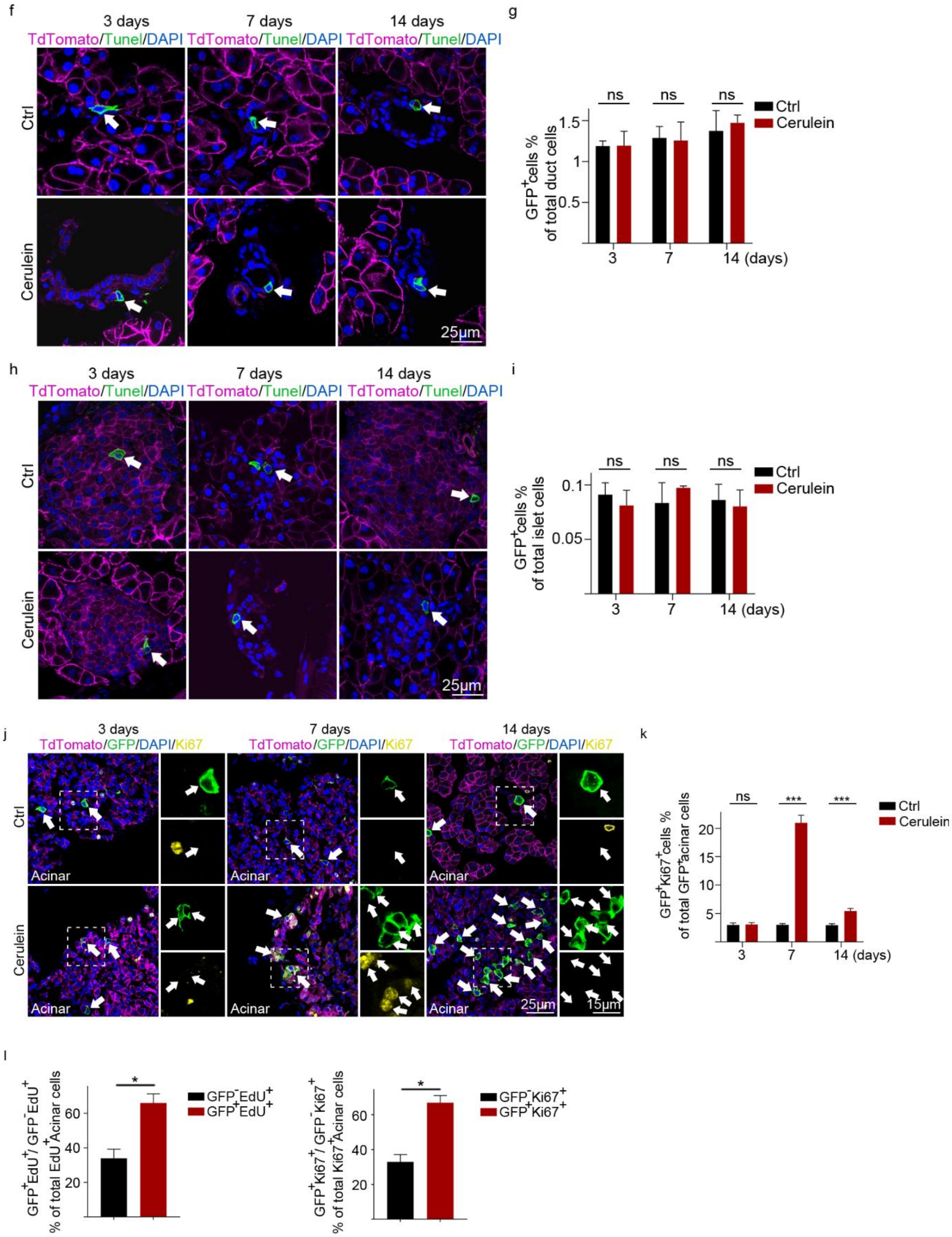
f
3 days
7 days
14 days
TdTomato/Tunel/DAPI
Ctrl
Cerulein
25μm
g
ns
Ctrl
Cerulein
GFP+cells % of total duct cells
3
7
14 (days)
h
3 days
7 days
14 days
TdTomato/Tunel/DAPI
Ctrl
Cerulein
25μm
i
ns
Ctrl
Cerulein
GFP+cells % of total islet cells
3
7
14 (days)
j
3 days
7 days
14 days
TdTomato/GFP/DAPI/Ki67
Ctrl
Cerulein
Acinar
25μm
15μm
k
ns
***
Ctrl
Cerulein
GFP+Ki67+cells % of total GFP+acinar cells
3
7
14 (days)
l
*
GFP-EdU+
GFP+EdU+
GFP+EdU+/ GFP-EdU+ % of total EdU+Acinar cells
GFP-Ki67+
GFP+Ki67+
GFP+Ki67+/ GFP-Ki67+ % of total Ki67+Acinar cells

Figure S4: Cerulein-induced pancreatitis in *SETD4-CreER*^{T2}*;Rosa26*$^{mTmG/+}$ mice.

(**a**) TUNEL staining of pancreas after 3, 7 or 14 days of cerulein treatments and of Ctrl. Ctrl: saline buffer treatment, n = 3 mice. Cerulein: cerulein treatment, n = 4 mice.

(**b**) Quantification of apoptosis acinar cells after 3, 7 or 14 days of cerulein treatments and the Ctrl.

(**c-e**) Hematoxylin-eosin staining of pancreas (c), PW/BW ratio, PW: pancreatic weight, BW: body weight (d) and pancreatitis score (e) after 3, 7 or 14 days of cerulein treatments and of Ctrl.

(**f** and **g**) Representative immunofluorescence (f) and quantification (g) of GFP recombinant cells in duct compartment after 3, 7 or 14 days of cerulein treatments and of Ctrl.

(**h** and **i**) Representative immunofluorescence (h) and quantification (i) of GFP recombinant cells in islet compartment after 3, 7 or 14 days of cerulein treatments and of Ctrl.

(**j** and **k**) Representative immunofluorescence for Ki67 (j) and quantification of GFP^+Ki67^+ cells (k) in the acinar compartment after 3, 7 or 14 days of cerulein treatments and of Ctrl.

(**l**) Quantification for proliferating GFP^+ and GFP^- cells by analyzing the proliferation marker EdU and incorporated Ki67.

All data are represented as mean ± SD. **p* < 0.05, ****p* < 0.001. ns: not significant. Arrows indicate recombinant GFP^+ cells (green). Nuclei were stained with DAPI.

Figure S5.

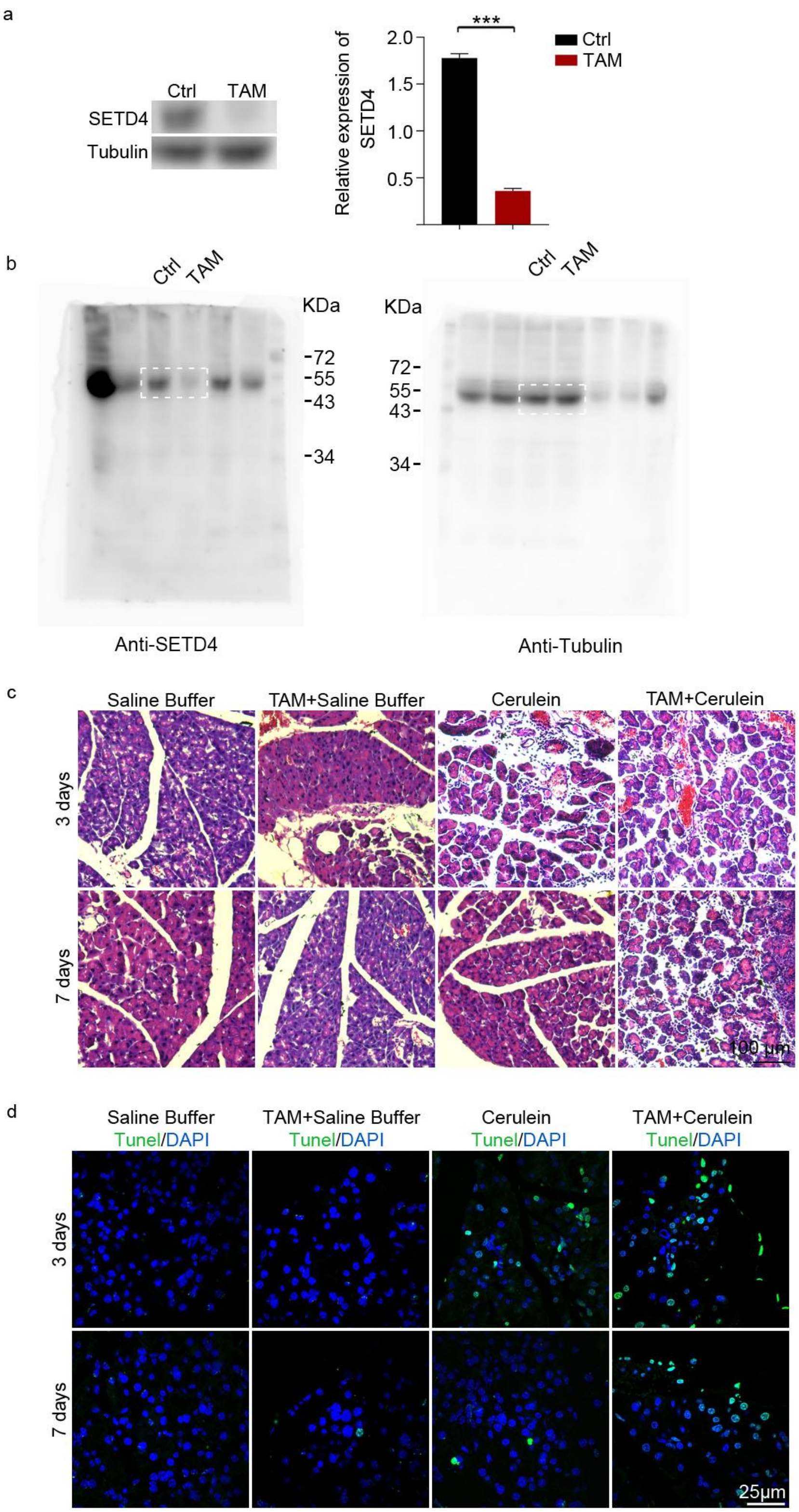

a
Ctrl
TAM
SETD4
Tubulin
Relative expression of SETD4
2.0
1.5
1.0
0.5
***
Ctrl
TAM
b
Ctrl
TAM
KDa
72
55
43
34
Anti-SETD4
Anti-Tubulin
c
Saline Buffer
TAM+Saline Buffer
Cerulein
TAM+Cerulein
3 days
7 days
100 μm
d
Saline Buffer
TAM+Saline Buffer
Cerulein
TAM+Cerulein
Tunel/DAPI
3 days
7 days
25μm

e
f
g

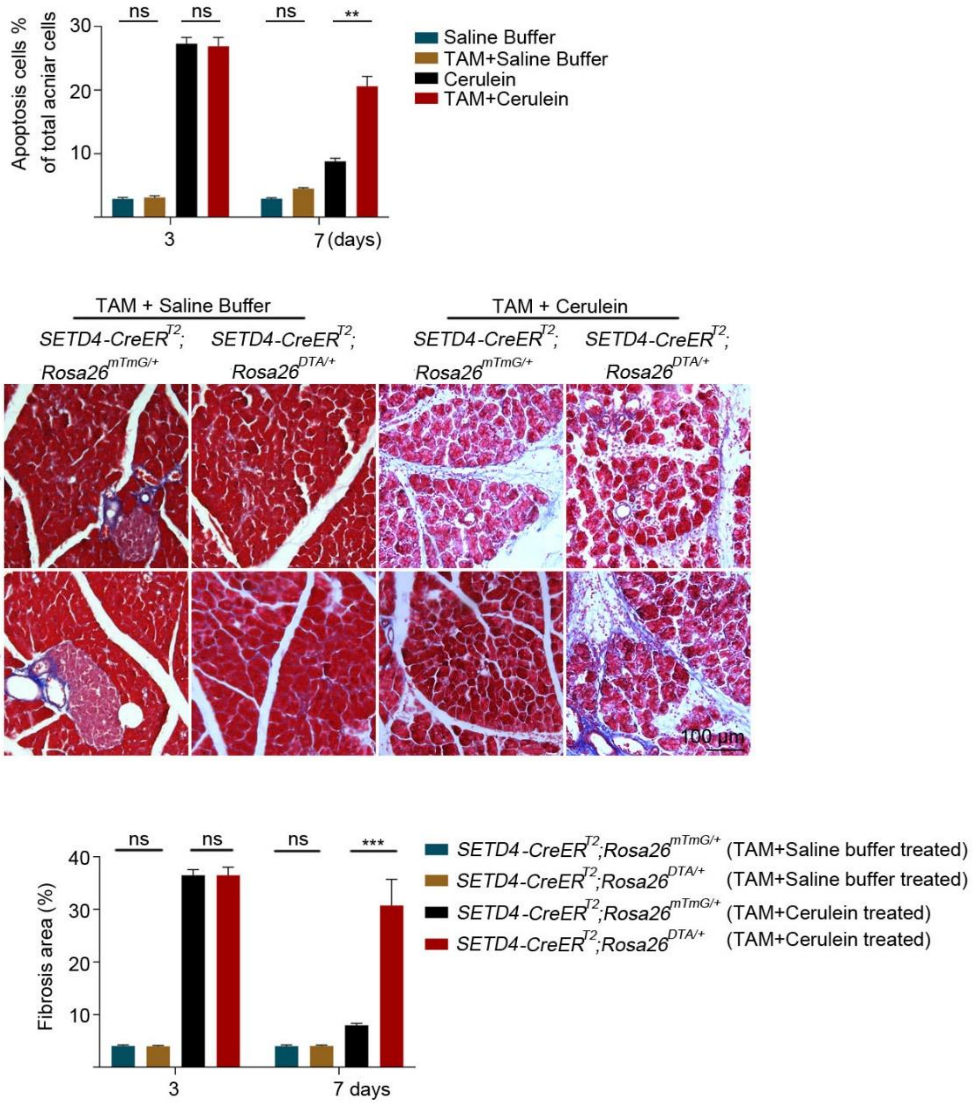

Figure S5: Analysis of SETD4 levels and histology and tunel staining of the acinar of *SETD4-CreER^{T2};Rosa26$^{DTA/+}$* mice after cerulein treatment.

(**a**) Western blot analysis and relative expression levels of SETD4 in the pancreas after 7 days of TAM-induction in *SETD4-CreER^{T2}*; *Rosa26$^{DTA/+}$* mice. Ctrl: saline buffer, TAM: TAM-induction.

(**b**) Full-length western blot image of SETD4 and Tubulin. The white dashed box indicates the target band.

(**c**) Representative of hematoxylin-eosin staining of pancreas after 3 or 7 days of TAM-induction and cerulein treatment in *SETD4-CreER^{T2}*; *Rosa26$^{DTA/+}$* mice.

(**d**) TUNEL staining of pancreas after 3, 7 or 14 days of cerulein treatments and of Ctrl in *SETD4-CreER*^{T2}; *Rosa26*$^{DTA/+}$ mice.

(**e**) Quantification of apoptosis acinar cells after 3,7 or 14 days of cerulein treatments and the Ctrl in *SETD4-CreER*^{T2}; *Rosa26*$^{DTA/+}$ mice.

(**f**) Masson's staining for pancreas tissue after the saline buffer and cerulein treatment. Pancreases were embedded in paraffin, sectioned to 6 μm thickness and stained with Masson; Scale bar, 100 μm.

(**g**) The fibrosis area occupied by blue-stained collagen was quantified using the ImageJ program. All data are represented as mean ± SD. $^{**}p < 0.01$, $^{***}p < 0.001$. ns: not significant. Nuclei were stained with DAPI.
